# Comprehensive identification of internal structure and alternative splicing events in circular RNAs

**DOI:** 10.1038/ncomms12060

**Published:** 2016-06-28

**Authors:** Yuan Gao, Jinfeng Wang, Yi Zheng, Jinyang Zhang, Shuai Chen, Fangqing Zhao

**Affiliations:** 1 Computational Genomics Lab, Beijing Institutes of Life Science, Chinese Academy of Sciences, Beijing 100101, China; 2 University of Chinese Academy of Sciences, Beijing 100049, China

## Abstract

Although previous studies demonstrated circular RNAs (circRNAs) does not exclusively comprise mRNA exons, no study has extensively explored their internal structure. By combining an algorithm with long-read sequencing data and experimental validation, we, for the first time, comprehensively investigate internal components of circRNAs in 10 human cell lines and 62 fruit fly samples, and reveal the prevalence of alternative splicing (AS) events within circRNAs. Significantly, a large proportion of circRNA AS exons can hardly be detected in mRNAs and are enriched with binding sites of distinct splicing factors from those enriched in mRNA exons. We find that AS events in circRNAs have a preference towards nucleus localization and exhibit tissue- and developmental stage-specific expression patterns. This study suggests an independent regulation on the biogenesis or decay of AS events in circRNAs and the identified circular AS isoforms provide targets for future studies on circRNA formation and function.

## Abstract (web summary)

Circular RNAs are increasingly understood to have important biological roles and have several subclasses. Here, the authors develop CIRI-AS to analyse sequencing data, identifying the prevalence of alternative splicing and circular RNA isoforms.

## Graphical abstract

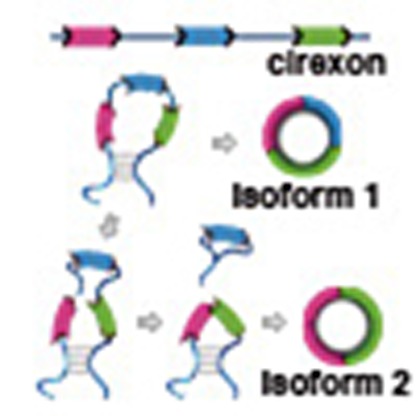

Over the past few years, it has been proved that circular RNAs (circRNAs) are ubiquitous in animals and some of them undertake essential biological functions^1^,^2^,^3^,^4^,^5^,^6^,^7^. Recently, new subclasses of circRNAs have been discovered in several studies. For example, Salzman *et al.*^8^ unexpectedly found a circRNA comprising a complete intron and flanking exons in gene *CAMSAP1*. Our previous study revealed the prevalence of non-exonic circRNAs containing intronic or intergenic circRNA fragments (ICFs) that are not expressed in known linear RNAs^9^. In a most recent report, Li *et al.*^10^ termed intron-retaining circRNAs as exon–intron circRNAs (EIciRNAs) and further proved that two EIciRNAs can interact with U1 small nuclear ribonucleoproteins and enhance the expression of their parental genes. Non-exonic circRNAs and EIciRNAs both contain sequences not present in mature messenger RNAs or other transcripts, which indicates that circRNAs does not exclusively comprise known exons but could have their specific internal compositions. Latest studies on biogenesis of circRNAs also revealed the association of the internal compositions with circRNA generation. By using customized circRNA expression vectors, Ashwal-Fluss *et al.*^1^ pointed out the importance of internal sequences for circularization efficiency of circRNAs with short flanking introns. A more recent report further proved that the collaboration between internal exons and the flanking intronic repeats is necessary in circRNA biogenesis^11^.

Compared with circRNA, structural characteristics and biogenesis of mRNA have been deeply studied. Alternative splicing (AS) is believed to be a key contributor to increased diversity of mRNAs and proteins in multicellular eukaryotes. It was estimated that about 95% of human genes with multiple exons are alternatively spliced^12^ and 2∼12 different isoforms can be generated for the majority of mammalian genes^13^. A wide range of biological processes, including apoptosis and sex determination, as well as many disease states, are correlated with genetic switches caused by AS events such as exon skipping (ES), alternative 5′ or 3′ splicing site (A5SS and A3SS) and intron retention (IR)^14^. Considering that both of mRNA and circRNA require the involvement of spliceosome during their formation^1^,^4^,^11^, it would be intriguing to explore (1) whether AS event is also prevalent in circRNA formation and (2) whether the AS events in circRNA are in the same pattern as in the corresponding mRNA. However, although previous reports discovered and investigated large amounts of circRNAs based on the recognition of back-spliced junctions (BSJs)^5^,^9^,^15^,^16^,^17^,^18^,^19^,^20^, there is no study extensively identifying and exploring internal components including AS events within circRNAs using high-throughput experimental or computational approaches, which largely restricts our understanding of circRNA biogenesis and function.

The difficulties first encountered in exploring such internal structure are attributable to a significant overlap of genomic localization of circRNAs with linear mRNAs. Our previous study showed that >95% of circRNAs have their linear counterparts in transcriptomic data^9^, which challenges a study aiming to differentiate and compare compositions between these two types of transcripts. On the other hand, the lower expression level of most circRNAs, which was estimated to account for ≤10% of corresponding mRNA expression^8^, further raises the susceptibility of an analysing approach to interference of linear transcripts and probably leads to biased estimation of the abundance of circRNA isoforms. To solve the above problems, we develop a novel algorithm for accurate detection of circRNA exons (cirexons) and AS events by analysing the spliced junction signatures of transcriptomic data.

In this study, we apply our algorithm along with long-read sequencing analysis and experimental validation to ten human cell lines, as well as 62 *Drosophila melanogaster* samples of distinct tissues or developmental stages, which is the first comprehensive investigation on cirexons and reveals the prevalence of AS events within circRNAs.

## Results

### Cirexon detection by a *de novo* algorithm

**Figure 1:**
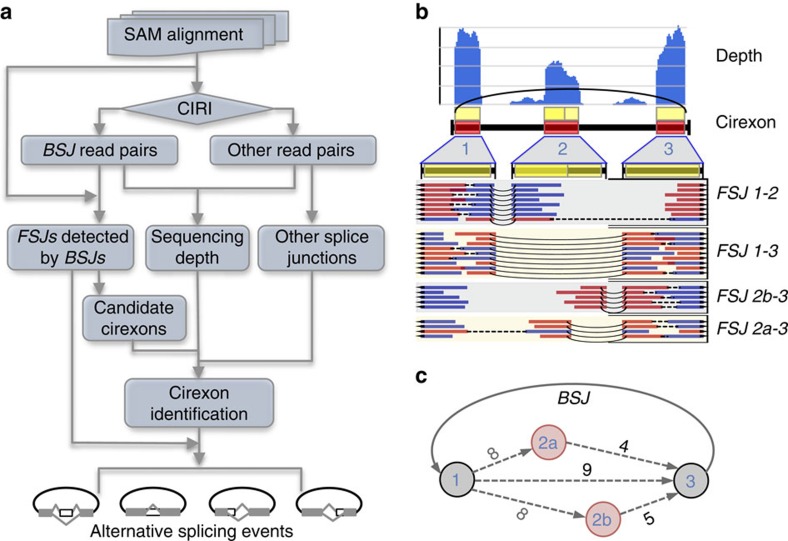
The algorithm and workflow of CIRI-AS. (**a**) The workflow of cirexon and AS detection. BSJ, back-spliced junction; FSJ, forward-spliced junction. (**b**) BSJ read pairs are peculiar to circRNAs and are thus used to identify splice junctions, indicating boundaries of cirexons. Red and blue bold lines indicate BSJ read pairs, which are connected by black dashed lines. Curved black lines indicate the FSJs present within circRNAs. (**c**) All possible routes are constructed using identified FSJs and cirexons to detect AS events within circRNAs. Red and grey nodes indicate alternatively spliced and constitutive cirexons, respectively. Dashed lines and numbers represent FSJs and supporting read pairs, respectively, in which grey numbers indicated that the FSJs are involved in multiple routes and supporting read pairs for each route are summed.

As an initial step to study circRNA internal structure, we developed a *de novo* algorithm for cirexon detection, named CIRI-AS. To circumvent undesired splicing signals from linear transcripts, this algorithm primarily focuses on BSJ read pairs peculiar to circRNAs provided by CIRI^9^ and uses split alignments of the BSJ read pairs provided by BWA-MEM^21^ as the major indicator of splicing events within circRNA (Fig. 1 and Supplementary Fig. 1). Briefly, CIRI-AS compares local alignment position and strand of each neighbouring segment pair within a BSJ read and its paired read, to recognize forward-spliced junctions (FSJs). Splicing acceptor of one upstream FSJ/BSJ and splicing donor of another downstream FSJ/BSJ with reasonable relative positions may indicate two ends of a cirexon. For each candidate cirexon, BSJ read pair coverage, sequencing depth variation and additional splice junctions indicated by non-BSJ reads are taken into account, to determine whether the candidate itself or in-between discontinuous segments are included in the circRNA. Details about cirexon detection algorithm are described in Methods.

We evaluated the efficiency of this new algorithm on cirexon detection using simulated circRNA-containing transcriptomic data. CIRI simulator^9^ was adapted to generate such data sets with various sequencing depths, insert length distributions and read lengths. As shown in Supplementary Fig. 2A,B, CIRI-AS had great performance on moderately and deeply sequenced circRNAs in the vast majority of simulated data sets. For example, sensitivity for circRNA with sequencing depth of 25-fold ranged from 72 to 79% in all four 100-bp libraries with different insert length distributions (Supplementary Fig. 2A). It has to be mentioned that the sensitivity above was calculated based on the whole detection pipeline and the loss of sensitivity indeed resulted from both circRNA and cirexon detection. For circRNAs successfully detected by CIRI, 80∼86% of cirexons could be predicted by CIRI-AS in the same data sets. In most cases, library insert length and sequencing read length did not affect the performance of CIRI-AS. However, CIRI-AS showed relatively low sensitivity for short read lengths, especially when sequencing depth was limited (Supplementary Fig. 2B).

**Figure 2:**
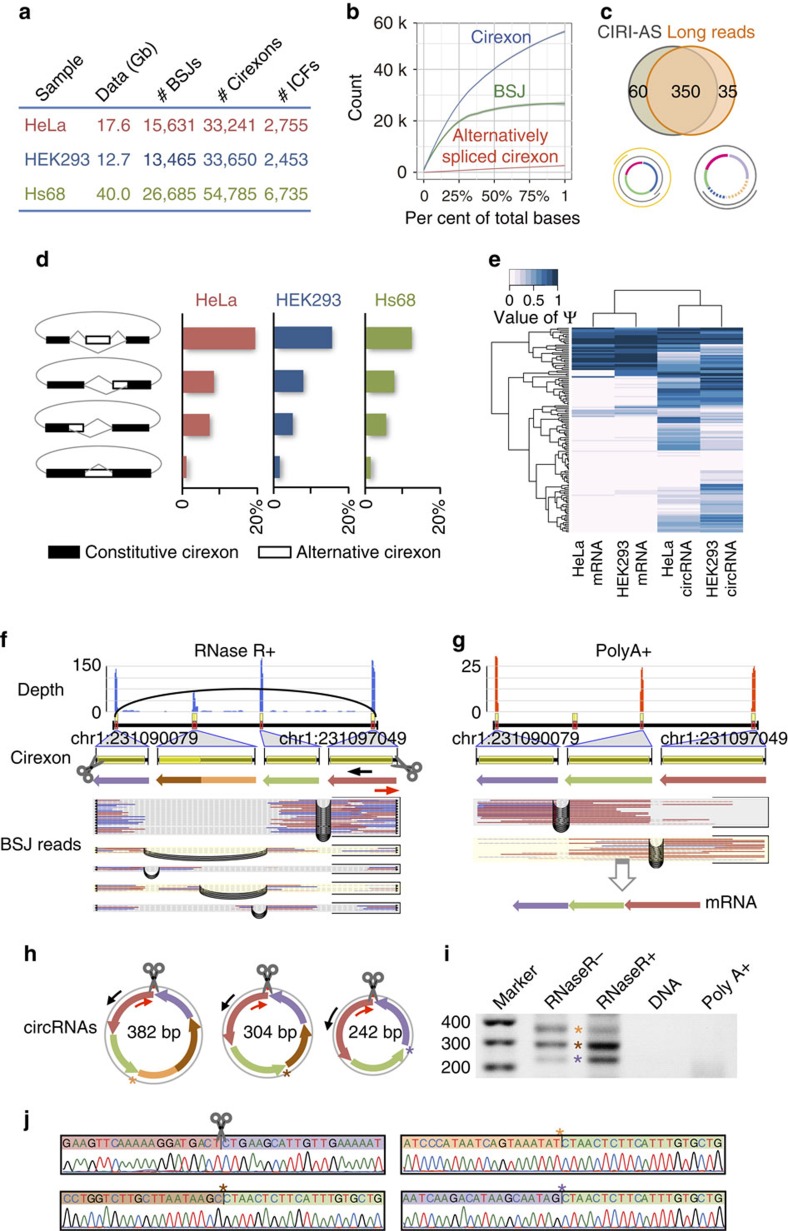
Cirexon and AS detection in three human cell lines. (**a**) BSJs, cirexons and ICFs detected in the three samples treated by RNase R. (**b**) Rarefaction analysis of detected cirexons, as well as corresponding BSJs and alternatively spliced cirexons in Hs68. (**c**) Overlap of cirexon detection of CIRI-AS using 100 bp paired-end reads and long reads. The bottom circular plots indicate two circRNAs and their cirexons validated by long-read sequencing (grey and orange spiral lines). Inner coloured solid-line curves indicate the cirexons detected by CIRI-AS, whereas the dashed-line curves represent the cirexons missed by CIRI-AS. (**d**) Percentage of circRNAs (≥20 BSJ reads) containing four types of alternatively spliced cirexons in the three samples: ES, alternative 3′-splicing site, alternative 5′-splicing site and IR. (**e**) Comparison of Ψ value for ES events between circRNA and mRNA in HeLa and HEK293 samples. (**f**–**j**) An experimentally validated example of alternatively spliced cirexons: ES and alternative 5′-splicing site within circRNA chr1:231,090,079|231,097,049. (**f**) Cirexons and splice junctions, as well as sequencing depth and corresponding BSJ reads, within the circRNA detected by CIRI-AS in RNase R-treated sample of HeLa cells. (**g**) Exons and splice junctions, as well as sequencing depth and corresponding sequencing read pairs, in poly(A)-selected sample of HeLa cells. (**h**) Positions of outward-facing primers and critical splice junctions on the three circular transcipts sharing the same BSJs. (**i**) The amplified fragments by RT–PCR corresponding to the three circular transcripts. (**j**) Sequencing chromatograms across the critical splice junctions of the PCR products.

To explore the internal structure of circRNAs, we applied CIRI-AS to deeply sequenced data sets generated in this and previous studies including transcriptomic data on human cell lines of HeLa, HEK293 and Hs68. In total, 212 Gb data based on RiboMinus RNA of samples with and without RNase R treatment for these three cell lines were used. In each data set, 3.9 × 10^3^∼5.5 × 10^4^ cirexons were detected (Fig. 2a and Supplementary Table 1). As RNase R specifically digests linear RNAs with free 3′ends and thus efficiently enriches circRNAs, samples treated by RNase R tend to generate deeper sequencing depths for circRNAs compared with those without RNase R treatment and thus facilitate cirexon detection. In four such RNase R-treated samples, we detected 3.3 × 10^4^∼5.5 × 10^4^ cirexons, corresponding to 1.4 × 10^4^∼3.0 × 10^4^ BSJs. Although most of the cirexons are known linear mRNA exons, 5.6∼16.8% of them are from intronic or intergenic regions. In addition, >10% of detected circRNAs in each sample contain such ICFs, consistent with our previous study^9^. We next selected the largest data set from Hs68 with RNase R treatment for a rarefaction analysis of detected cirexons and corresponding BSJs. In contrast with a near saturation of BSJ detection, identified cirexons showed a steady increase along with the accumulation of sequencing bases (Fig. 2b), which indicated that comprehensive identification of cirexons necessitates much more sequencing data compared with BSJ detection.

To validate the cirexons discovered by our method, in particular for their boundaries, we generated paired-end 250 bp RNA-seq data for the HeLa sample with an estimated insert length of 450 bp. Using this new long-read data set, we reconstructed the complete sequence of circRNAs and used them to compare with cirexons detected by CIRI-AS on the PE100 RNA-seq data of the same sample. Among the 173 circRNAs detected in both data sets, 350 out of 385 cirexons present in the long-read data could be predicted by CIRI-AS (Fig. 2c and Supplementary Fig. 3), demonstrating the high reliability of discovered cirexons. However, the remaining 35 cirexons (∼9% of 385) found in the long-read data were not detected by CIRI-AS (Fig. 2c). Scrutiny of these cirexons revealed that most of them were from very low abundant circRNAs and the rareness of BSJ reads largely accounted for the inability of detection (Supplementary Fig. 4). For example, CIRI-AS found the two cirexons flanking the circular junction of circRNA chr13:111,857,636|111,870,227 by recognizing its only three BSJ read pairs, but missed the cirexon in between (Supplementary Fig. 4A).

### AS events detection and validation within circRNAs

Besides the cirexons discovered by both methods and those only by the long-read data, there were 60 cirexons exclusively predicted by CIRI-AS (Fig. 2c). These predictions and the corresponding FSJs could not be ruled out by applying higher thresholds such as supporting read count and mapping quality, suggesting they are not false-positive predictions by CIRI-AS. A manual scrutiny of the BSJ reads of these cirexon predictions revealed that most of them were alternatively spliced cirexons, which were only present in some of the circular transcripts but absent in others sharing the same BSJs (Supplementary Fig. 5). For example, CIRI-AS predicted a 50-bp ICF within the circRNA chr9:134,312,007|134,314,448 and there were 14 and 15 supporting reads of FSJs for the 3′- and 5′-end of this cirexon, most of which had no other alignment along the human genome and showed high mapping qualities (Supplementary Fig. 5D). Although the circular transcript with skipped ICF was captured by both methods, the transcript containing this ICF was only found in the deep sequencing PE100 data.

To comprehensively identify various types of AS events in circRNAs, we developed a new algorithm based on reconstructing cirexon routes as well as clustering alternatively spliced cirexons, which was implemented in CIRI-AS. For example, three routes representing three potential circular transcripts contain a cirexon 2a, its equivalent cirexon 2b or neither, respectively (Fig. 1c). As there is a transcript splicing both cirexon 2a and cirexon 2b out, it can be classified as an ES event, while simultaneously cirexon 2a and cirexon 2b can have alternative 5′- or 3′-splicing site. Details about cirexon detection algorithm are also included in Methods.

We then used the above algorithm to detect AS events in samples of Hs68, HeLa and HEK293 treated by RNase R. Strikingly, all four types of AS events could be found within circRNAs in all of the three samples (Supplementary Tables 2–4 and Supplementary Fig. 6). For example, ES is the most common AS type in circRNAs and we found skipped cirexons within 735, 428 and 583 circRNAs of the three cell lines, respectively, which account for 2.7∼4.3% of total circRNAs found in these samples. A3SS and A5SS are also major circular AS types, both of which occur in >1% of detected circRNAs. Our results further revealed that A3SS has a higher frequency than A5SS in all of three cell lines (1.8% versus 1.2%, 1.4% versus 1.1% and 2.1% versus 1.4%), which is similar to AS events in mRNAs reported previously^22^. Of the four AS types, IR is the rarest one, accounting for 0.1∼0.3% of total circRNAs.

It has to be mentioned that rarefaction analysis on Hs68 sequencing data showed that detection of alternatively spliced cirexons was far from saturation (Fig. 2b), suggesting that rare circular AS isoforms remain to be sequenced or discovered and thus the frequencies of AS events calculated based on all detected circRNAs were likely to be underestimated. To better estimate frequencies of AS events, especially for relatively abundant circRNAs that may undertake biological functions, we set a simple cutoff for circRNAs using BSJ read count, which was often used as a rough estimate of sequencing depth or abundance of circRNA in previous reports^8^,^23^,^24^. When such cutoff was set to 20, we got 1,262, 1,952 and 3,910 circRNA loci in HeLa, HEK293 and Hs68 cell lines, respectively, of which a much higher proportion had alternatively spliced isoforms (Fig. 2d). For example, 19.2, 15.3 and 12.2% of the circRNA loci in the three cell lines have ES isoforms, respectively, and even the rarest AS-type IR occurred in >1% of the total circRNAs. Such high frequencies strongly suggest that AS events not only occur in mRNAs but also are prevalent in circRNAs. We also observed ten examples of AS events with altered density of microRNA binding sites in circRNAs, which suggest their potential roles in gene regulation (Supplementary Fig. 7 and Supplementary Table 5).

To further verify that these predicted AS events take place in circRNAs, we performed experimental validation for randomly selected 25 circRNA loci in HeLa samples (Supplementary Table 6). Each of these circRNAs was predicted to contain at least one type of alternatively spliced cirexons. We designed outward-facing primers to amplify fragments containing BSJ and alternatively spliced cirexons, and performed Sanger sequencing on the products. As a result, 22 loci of them, including 12 loci for ES (Supplementary Fig. 8), seven loci for A3SS and A5SS (Supplementary Figs 9 and 10), and three loci for IR (Supplementary Fig. 11), were successfully validated. As no corresponding products were amplified from negative control of total DNA or poly(A)-selected samples, these validations proved the reliability of our predictions and also provided solid evidence for widespread occurrence of AS events within circRNAs. To further confirm the resistance of predicted circular AS isoforms to RNase R, we performed quantitative reverse transcriptase–PCR (qRT–PCR) for nine AS isoforms in four circRNA loci and found that all of them were significantly more resistant compared with linear mRNA (Supplementary Fig. 12). In addition, some AS events detected by CIRI-AS could be further confirmed by long-read sequencing data (Supplementary Fig. 13). Unlike 100-bp short reads, these long reads could record the full length of the AS isoforms, including both BSJ and all cirexons. Interestingly, we also found that some of the validated alternatively spliced events are circRNA specific when comparing them with mRNA AS events in the corresponding poly(A)-selected sample. For example, three circular isoforms were detected in chr1:231,090,079|231,097,049 of gene *TTC13* (Fig. 2f,h–j), whereas no AS event was present in the corresponding mRNA transcript (Fig. 2g). In detail, although exon 2, exon 3 and exon 5 of *TTC13* were found in all of the mRNA and circRNA isoforms, exon 4 and a cirexon with alternative 5′-splicing site of exon 4 are only present in the 382- and 304-bp circRNA isoform, respectively.

### Estimation of relative abundance for AS events in circRNAs

We next asked whether the above distinction between AS patterns of circRNAs and mRNAs is occasional or pervasive. The value of ‘percentage spliced in' (Ψ), which was used to represent relative abundance of AS event in previous studies on mRNA^13^, provided an appropriate indicator for comparison between circRNA and mRNA AS events. In CIRI-AS, Ψ value was estimated based on specific type of AS event and corresponding supporting read count of related FSJs. We also adopted a correction method by assessing the influence of library insert length distribution on the abundance estimation to eliminate bias caused by ‘anchored' position of BSJ reads. Details about relative abundance estimation of AS in circRNA are included in Methods.

To test the accuracy of Ψ estimation in our approach, we simulated transcriptomic data containing skipped cirexons in circRNAs with various sequencing depths and relative abundances. As expected, the Ψ estimation simply based on relative reads density showed a slight bias towards underestimation in all data sets with a simulated Ψ value of 0.25, 0.5 and 0.75 (Supplementary Fig. 14A). As the estimation was based on BSJ read pairs, which represent only a subset of sequencing reads of circRNAs, the bias it caused is systematic and cannot be improved by merely increasing sequencing depth (Supplementary Fig. 14B). However, after taking library insert length distribution into consideration and making corresponding corrections, this systematic bias was effectively eliminated and our algorithm showed a better performance for Ψ estimation. We also used qRT–PCR to quantify relative abundance of circRNA AS isoforms that we detected in HeLa cells (Supplementary Table 7). As shown in Supplementary Fig. 15, relative abundances estimated by CIRI-AS and qRT–PCR were highly consistent with each other for all nine AS isoforms, which further demonstrated the reliability of CIRI-AS on relative abundance estimation.

We next pursued our investigation on relative abundance of ES in HeLa and HEK293 cell lines. The values of Ψ were estimated for all detected skipped cirexons in both of the cell lines using the above algorithm and were compared with the expression of corresponding mRNA transcripts in poly(A)-selected samples. The result showed a dramatic difference on splicing efficiency of skipped exon between circRNA and mRNA (Fig. 2e). We applied statistical tests based on a β-binomial model and found that 78.5% of the ES events have a significantly different Ψ value in the two types of RNA (*P*-values <0.05), of which more than half had a much larger Ψ value (ΔΨ >0.4) in circRNAs compared with the corresponding mRNAs in at least one cell line. A large proportion of alternatively skipped exons in circRNAs were never or seldom expressed in the corresponding mRNAs. A hierarchical clustering based on Euclidean distances of the Ψ values showed that ES within circRNAs of different cell lines have more similar relative abundances compared with that in the corresponding mRNAs of the same cell line (Fig. 2e), which suggest that AS events within circRNAs might be under regulation independent to mRNA splicing.

Among these with higher relative abundance in circRNAs compared with mRNAs in HeLa and HEK293, 54% could be classified as ICFs that were never found in mature mRNAs transcripts, whereas the remaining 46% were mainly from known transcripts annotated as ‘protein coding', ‘processed transcript' or ‘nonsense-mediated decay' (NMD) in Gencode (Supplementary Fig. 16). NMD pathway can specifically degrade mRNA transcripts with premature stop codons^25^. We found that the cirexons with much higher Ψ values (ΔΨ >0.4) in circRNAs than in mRNAs were more likely to contain premature stop codons than the remaining alternatively skipped cirexons (64.5% versus 35.0%). To explore whether the above distinct expression pattern between circRNA and mRNA results from mRNA-specific decay pathway, we also analysed a transcriptome data set of mouse immune dendritic cells with 10-min labelling pulse of 4-thiouridine, which is highly enriched with newly transcribed RNA including NMD transcripts^26^. By applying CIRI-AS on the 4-thiouridine-labelled data, we observed a subset of skipped cirexons still showed significantly higher relative abundance in circRNA than the corresponding mRNA (Supplementary Fig. 17).

**Figure 3:**
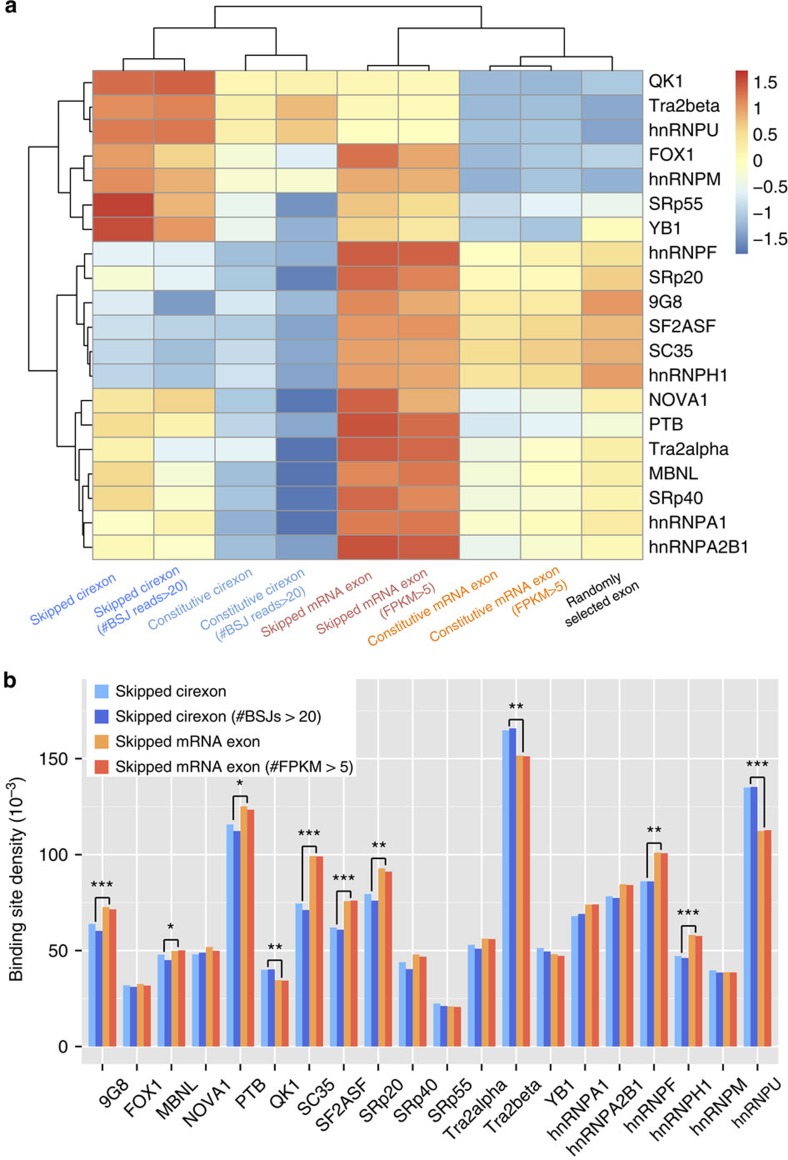
Binding site density of splicing factors in transcribed exons of HeLa cells. (**a**) Binding site density of splicing factors in skipped/constitutive cirexons, skipped/constitutive mRNA exons and randomly selected annotated exons, as well as their adjacent regions. The average binding density was calculated as the percentage of binding sites in the whole length of the sequence for prediction and shown in the heatmap after *Z*-score normalization. (**b**) Comparison for the above density between skipped cirexons and skipped mRNA exons with the corresponding statistical significances of Mann–Whitney *U*-test. *P-value <0.05; **P-value <0.01; ***P-value <0.001.

It has been reported that several splicing factors and RNA-binding proteins are involved in regulating the biogenesis of circRNAs, such as MBNL^1^, QKI^27^, heterogeneous nuclear ribonucleoprotein and SR proteins^28^. To further explore the potential mechanism of the independently regulated AS events, we investigated binding site density of known splicing factors and RNA-binding proteins in skipped/constitutive cirexons and skipped/constitutive mRNA exons, as well as their adjacent regions using existing algorithms^29^,^30^. As shown in Fig. 3a and Supplementary Fig. 18, most binding sites are significantly enriched in skipped mRNA exons or skipped cirexons compared with the corresponding constitutive exons and randomly selected annotated exons (also see Supplementary Table 8 and Supplementary Fig. 19; Mann–Whitney *U*-test: *P*-values <0.05 after false discovery rate (FDR) correction). Interestingly, the regulatory factors with enriched binding sites in skipped cirexons are distinct from those in skipped mRNA exons. For example, binding sites of QKI, Tra2β and heterogeneous nuclear ribonucleoprotein-U are significantly more enriched in skipped cirexons compared with skipped mRNA exons, whereas other seven splicing factors showed an opposite trend (Fig. 3b; Mann–Whitney *U*-test: *P*-values <0.05 after FDR correction). Consistent with a recent study, in which the production of over one-third of abundant circRNAs were found to be regulated by the splicing factor QKI^27^, our analyses indicate that AS events in circRNAs may also have their own preference on regulatory factors. Taken together, the above results suggest an independent regulation of circRNA biogenesis or decay may account for the distinct expression pattern of alternative spliced exons between circRNAs and mRNAs.

### Localization and characterization of IRs in circRNAs

**Figure 4:**
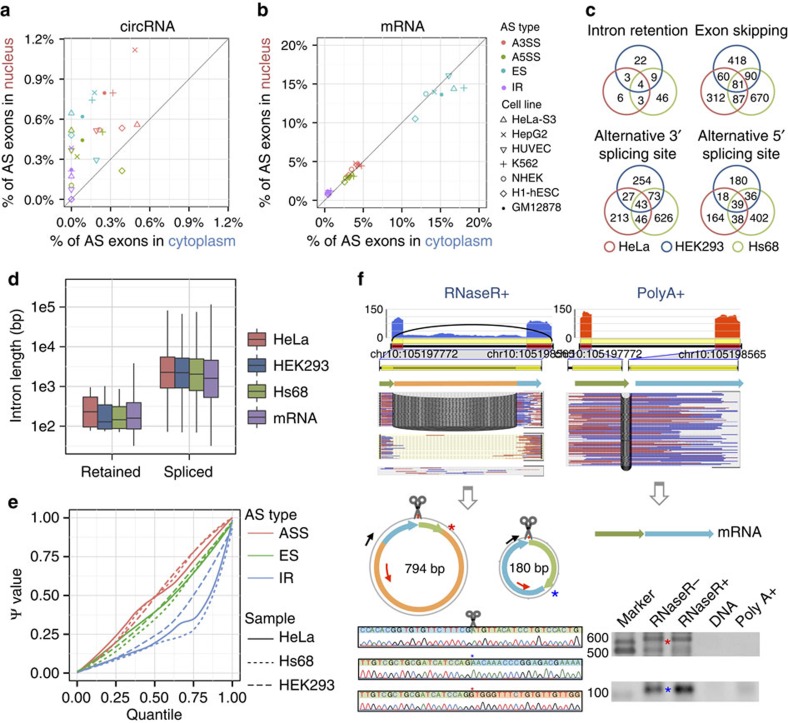
Features of four types of AS events in circRNAs. (**a**–**b**) Four types of AS events are enriched in the nucleus. Transcriptomic data sets (poly(A)− and poly(A)+) from cytoplasm and nucleus RNA samples of seven cell lines in ENCODE were used to predict AS events. (**a**) Percentage of alternatively spliced cirexons in all detected cirexons within circRNA. (**b**) Percentage of alternatively spliced exons in all detected exons of mRNA. (**c**) Overlap of IR, ES and alternative 3′- or 5′-splicing site in HeLa, HEK293 and Hs68 samples treated by RNase R. (**d**) Comparison of retained intron length with other intron length within circRNA, as well as those in mRNA. (**e**) Sorted Ψ values of IR and other AS events in HeLa, HEK293 and Hs68. (**f**) An experimentally validated example of alternatively spliced cirexons: IR within circRNA chr10:105,197,772|105,198,565. Cirexons and splice junctions, as well as sequencing depth and corresponding BSJ read pairs, within the circRNA detected by CIRI-AS in RNase R-treated sample of HeLa cells. Exons and splice junctions, as well as sequencing depth and corresponding sequencing reads, in poly(A)-selected sample of HeLa cells. Different outward primers were designed and RT–PCR was performed separately for each isoform. Sanger sequencing was used to validate one BSJ junction and two FSJ junctions.

It was reported that intron-retaining circRNAs may function as nucleus-localized transcription activators^10^. To systematically investigate the localization preference of alternatively spliced circRNAs, we applied CIRI-AS to both nucleus and cytosol poly(A)-/RiboMinus RNA-transcriptomic data in ENCODE including three cancer cell lines (HeLa-S3, HepG2 and K562) and four non-cancer lines (H1-hESC, GM12878, HUVEC and NHEK). We first compared the percentage of alternatively spliced circRNAs between the two cellular localizations and found that all four AS events in circRNAs have a preference towards nucleus localization in the seven cell lines (Fig. 4a and Supplementary Fig. 20; paired *t*-tests: *P*-values <0.05). For example, HepG2 cytosol and nucleus data set have comparable data sizes (24.5 Gb versus 30.1 Gb), but much more proportion of alternatively spliced circRNAs were detected in the nucleus data set. By contrast, mRNAs showed no localization preference for three types of AS events including SE, A3SS and A5SS in the corresponding poly(A)+ transcriptomic data (Fig. 4b), although higher proportion of IR events in mRNA was detected in the nucleus (Supplementary Fig. 21; paired *t*-test: *P*-value <0.01). Indeed, it has been reported in previous studies^31^,^32^ that mRNAs containing retained introns locate in both the nucleus and cytosol, but more frequently in the nucleus. However, circRNA IR showed a more extreme tendency, that is, all of the 23 retained introns detected in the seven cell lines come from the nucleus (Supplementary Fig. 21 and Supplementary Table 9). A further survey on the cytosol transcriptomic data confirmed the existence of the 23 corresponding circular isoforms with these introns spliced out. For example, one isoform of gene *CLK1* containing exon 8 and exon 9 is observed in both the nucleus and cytosol, whereas the other isoform with intron 8 retained only exists in the nucleus and these IR events could not be detected in poly(A)-selected data sets of the nucleus or cytosol according to our analysis and Cufflinks^33^ (Supplementary Fig. 22).

We next focused on IR events detected in the samples of HeLa, HEK293 and Hs68 treated by RNase R. Although IR is the rarest AS type in circRNAs, we found that >20% (19 out of 93) of retained introns are shared in at least two cell lines and 4 of them exist in all of the 3 cell lines (Fig. 4c), which strongly suggest they are not random products of mis-splicing events. To further characterize IR in circRNAs, we investigated their lengths and relative abundances. Retained intron lengths were about two orders of magnitude smaller compared with other spliced introns in circRNAs and with those in linear transcripts (Mann–Whitney *U*-test, *P*-value <0.001), but showed no significant difference with mRNA retained introns (Fig. 4d). As to relative abundance, the circular transcripts containing retained introns tended to have lower expression levels compared with corresponding alternatively spliced transcripts with the intron spliced out in the same sample (Fig. 4e). Similar with skipped exons peculiar to circRNAs, we also detected and validated retained introns within circRNAs but not present in corresponding mRNAs (Fig. 4f and Supplementary Fig. 11C).

### Tissue- and developmental stage-specific circRNA AS pattern

To study AS events not only in cultured cell lines but also in different tissues and developmental stages, we applied our algorithms to 103 transcriptome data sets from 62 samples of *D. melanogaster* including developmental stages such as embryo, larva, pupa and adult, as well as various tissues and cell lines^24^. The detection of circRNAs indicated that much fewer circRNAs exist in fruit fly compared with that in human. For example, we found 268∼705 circRNAs in each cultured cell line of fruit fly, whereas 2,532∼6,606 circRNAs were identified in RiboMinus samples of Hs68, HeLa and HEK293 with comparable data amounts using the same criteria (Supplementary Tables 1 and 10). However, such difference reduced after normalization by gene length and exon number of the two species. Our algorithm detected previously unpredicted structures within circRNAs in fruit fly. For example, 4,481 cirexons were recognized from the top 2,000 most abundant circRNAs in all sequencing data, of which ∼21% (939 cirexons) were classified as ICFs according to the latest version of Ensembl annotation for *D. melanogaster*. Moreover, a further survey for AS events showed that ES, A3SS, A5SS and IR were also prevalent in fruit fly, with 303 circRNAs having multiple isoforms.

**Figure 5:**
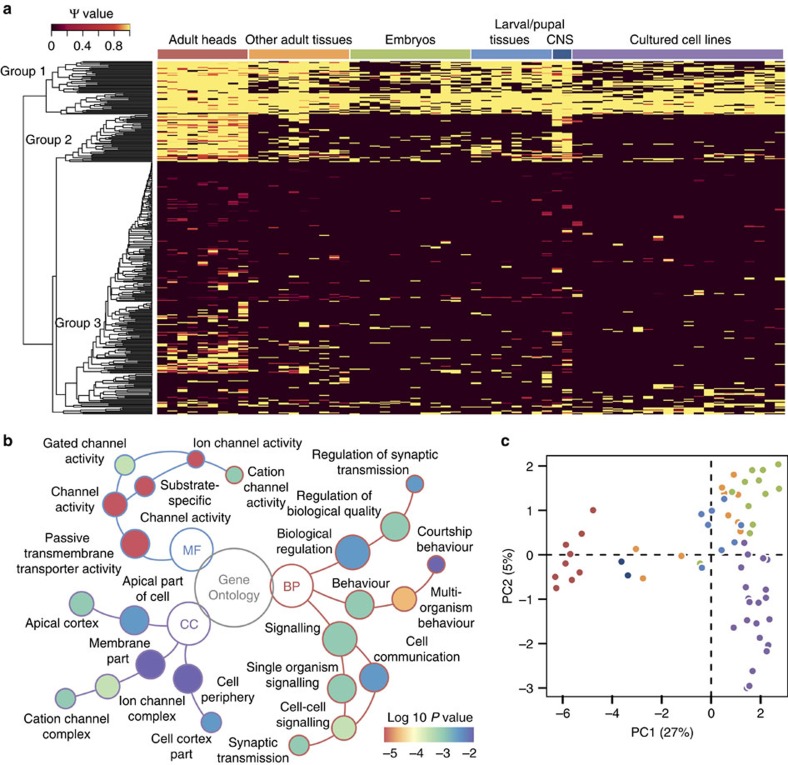
CircRNA AS events in 62 samples of *D. melanogaster.* (**a**) Relative abundance of detected AS cirexons in all of the 62 samples. The cirexons can be clustered into three distinct groups according to their Ψ values in the tissues and cell lines. Yellow denotes cirexons with Ψ value equal to 1 and dark brown denotes cirexon with Ψ value equal to zero or no expression/detection. (**b**) Gene Ontology enrichment analysis for parental genes of alternatively spliced circRNAs in adult heads and laval/pupal CNS. BP, biological process; CC, cellular component; MF, molecular function. (**c**) Principal components analysis based on the Ψ values of the AS cirexons in each sample. Each dot represents a sample and its colour corresponds to the sample type on the top of **a**.

We next asked whether these identified AS events within circRNAs were associated with specific tissues or development stages. The relative abundance of alternatively spliced cirexons was calculated and depicted in a heatmap. As shown in Fig. 5a, these cirexons could be clustered into three distinct groups according to their Ψ values in the 62 samples. The first group displayed an extensive distribution in almost all samples and the third group of cirexons sporadically existed in certain samples, whereas the second group specifically expressed in nervous systems such as dissected adult heads and larval/pupal central nervous system (CNS). We thus performed Gene Ontology over-representation test for parental genes of alternatively spliced circRNAs in adult heads and laval/pupal CNS. In addition to the gene sets relating to neural functions revealed in the previous study^24^, our result suggested that the genes of these circRNAs may be involved in other processes and functions, including behaviour and channel activity (Fig. 5b). Principal component analysis based on all alternatively spliced cirexons explained ∼32% of the total transcriptional variance in the first two principal components, distinguishing adult heads as the most divergent group. Similarly, a clear separation was also observed in other tissues or cell lines (Fig. 5c and Supplementary Figs 23 and 24). We also investigated AS events in mRNA using three corresponding poly(A)-selected data sets and found a distinct AS pattern compared with circRNAs (Supplementary Fig. 25). Considering that mRNA AS events were extensively reported to control genetic switches related to various developmental and cellular processes^34^, the close association between circRNA AS events and specific samples also suggested their potential roles in regulatory transitions in animals.

## Discussion

Although previous studies reported several pipelines and algorithms for identification of circRNAs, all of them focused on circular junction that was believed to be the main feature of circRNAs. Although in-depth investigation on circRNAs such as binding site analysis and functional validation calls for accurate information on internal compositions, few methods or techniques are currently available. Experimental approaches such as RT–PCR can provide precise sequence for a specific circRNA but are not applicable to high-throughput detection or analysis. Another common way of simply combining all known mRNA exons in a sequential order as putative full-length circRNA is indeed derived from an unsupported assumption of the same sequence usage for circRNA and mRNA, and may result in misunderstanding in subsequent analyses. By contrast, the algorithm we introduced in this study is based on spliced junction signatures of transcriptomic data rather than any *a priori* assumptions, and thus realizes *de novo* and high-throughput detection of internal components of circRNAs.

Among spliced junction signatures, our algorithm lays emphasis on those from BSJ read pairs instead of all sequencing reads. This strategy has multiple impacts on elaborating the internal structure of circRNAs. First, it facilitates precise cirexon identification without interference from mRNAs or other linear RNAs, which is an indispensable advantage for our subsequent investigation on circRNA internal components. Second, less abundant circRNAs with few BSJ reads may result in relatively low sensitivity on internal component detection. Therefore, deep sequencing of transcriptomes is a prerequisite for comprehensive identification of cirexon and AS events. Third, it may lead to a systematic bias on relative abundance estimation of circular isoforms due to biased sampling of BSJ read pairs along circRNA isoforms. Our algorithm can deal with this problem by employing a new correction approach as described in the Methods. Although whole sequence prediction and accurate relative abundance estimation of circular isoforms with complicate AS events still remain to be solved, our algorithm represents the first and optimal solution in current short-read sequencing, as well as a promising direction for circRNA methodology.

**Figure 6:**
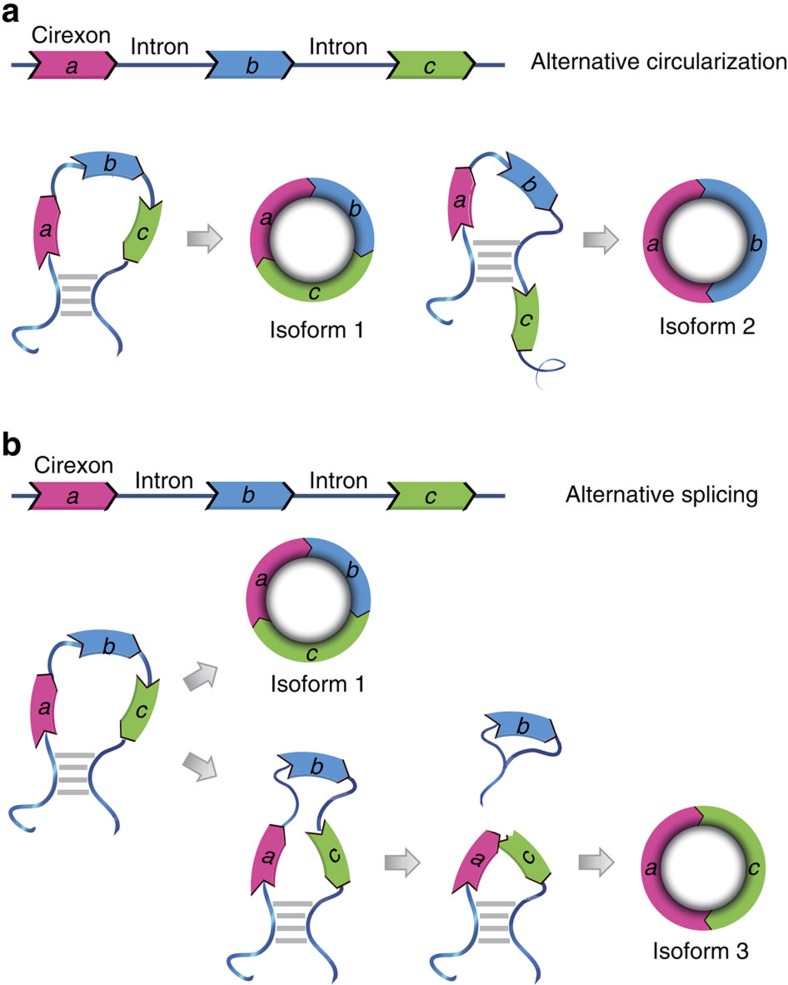
Both AC and AS contribute to the diversity of circRNAs. (**a**) Back-splicing donors differ between the two AC isoforms, which can be mediated by complementary sequences within flanking introns of exon a/c and exon a/b, respectively. (**b**) The two AS isoforms share the same back-splicing acceptor and donor but differ in their internal structure.

Owing to the presence of canonical GT/AG splicing sites in back-splicing, spliceosome that mediates AS and contributes to the diversity of both mRNAs and proteins in animals is also widely believed to be involved in circularization^11^,^28^,^35^. Several reports have characterized cases of multiple circular isoforms produced from a single gene but with different pairs of splicing acceptors and/or donors^4^,^8^, which were named as alternative circularization (AC) isoforms^36^ and considered as the key contributor of circRNA diversity (Fig. 6a). In this study, we, for the first time, demonstrated the prevalence of circular AS isoforms sharing the same splicing acceptor and donor, which represents another source of circRNA isoforms largely neglected before (Fig. 6b). Although AC isoforms generate different BSJ reads and therefore can be identified by existing circRNA detection algorithms, the circular AS isoforms described in this study were cryptic and difficult to detect, for they usually possess identical BSJ. Previous studies revealed that complementary sequences or protein-binding sites within flanking introns can mediate AC^27^,^36^, but these models still cannot adequately explain the biogenesis of circular AS isoforms sharing the same flanking introns but distinct in internal compositions, which strongly suggests that the biogenesis of circRNAs is more complicate than we previously thought.

Although it is widely believed that circRNAs are a class of noncoding RNAs^4^,^5^,^23^ and their expressions show little correlation with linear RNAs^8^,^37^ (Supplementary Fig. 26), previous reports often presume that circRNAs constitutively contain mRNA exons in their circles. The discovery of non-exonic circRNAs and EIciRNAs, however, largely challenged the assumption of the same tendency for circRNA and mRNA on sequence usage. In this study, the comparison on AS further revealed a significant distinction between circRNA and mRNA, and provided another layer of evidence for dissimilarity of internal composition of these two classes of RNAs. We found that some AS events can only exist in circRNAs but not in corresponding mRNAs and a large proportion of skipped cirexons in circRNAs have a significantly different (often larger) relative abundance compared with their counterpart in the corresponding mRNAs. These alternatively spliced cirexons represent a class of neglected targets for further study on circRNA biogenesis, regulation and novel function.

## Methods

### Overview of CIRI-AS

CIRI-AS is a novel algorithm for detecting internal components of circRNAs based on split alignment of BSJ read pairs and distribution of sequencing depth. It provides a solution to the problem that currently no high-throughput method is available for understanding internal components as well as AS within circRNAs. CIRI-AS can be used for organisms with or without annotation. It is also applicable to samples with or without RNase R treatment and efficient for most read lengths commonly used at present. CIRI-AS is implemented in Perl and has been extensively tested on Linux and Mac OS X. CIRI-AS is packaged with CIRI and CIRI simulator described in simulated data and is available at https://sourceforge.net/projects/ciri/.

### Input data for CIRI-AS

CIRI-AS requires three types of input data: reference genome formatted in FASTA, circRNA list generated by CIRI^9^ or other tools in a given format, and Sequence Alignment/Map (SAM) generated by BWA-MEM^21^ for the data set to be processed. The circRNA list provides CIRI-AS with the circRNAs to be analysed and the corresponding BSJ read pairs, and the SAM records local alignments for each read pair in the data. An optional input is GTF-formatted annotation of the reference genome. When the annotation is provided, distribution of insert lengths can be calculated and used for further correction of estimated Ψ values in AS events. In addition, the genome annotation facilitates the identification of intronic fragments or ICFs from cirexons predicted by CIRI-AS.

### Detection of FSJ and candidate cirexons using BSJ read pairs

As the BSJ read pairs of a circRNA have been identified to be indicators of the circular junction by CIRI or other tools, split alignments of the read pairs can provide confident evidence on FSJs within a circRNA. For each BSJ read pair, CIRI-AS collects all local alignments and sorts them according to their corresponding segment positions in the read pair. By comparing alignments of each neighbouring segment pair in a single read on both strand and position, CIRI-AS finds candidate FSJs and estimates position ranges of them on the reference genome. All BSJ read pairs indicating the same candidate splicing are then clustered for further filtrations, in which CIRI-AS searches boundaries of the estimated position ranges for potential splicing signals and decides the rationale of local alignment positions for other segments of the read pairs, to remove false positives. Accurate positions of splice donor and acceptor are determined according to detected splicing signals. The splice donor and acceptor of different splice junctions are then paired with each other to indicate two ends of a candidate cirexon.

### Cirexon detection using sequencing depth and non-BSJ reads

Although BSJ read pairs are reliable indicators of splice junctions within circRNAs, they are often restricted by abundance of circRNAs, read length and distribution of library insert length, and thus may not cover the whole circRNA in various occasions (Supplementary Fig. 1C). When BSJ read pairs failed to cover a FSJ, a fragment comprising two cirexons and the intron in between would be regarded as a cirexon candidate. To rule out such false candidates, CIRI-AS next checks the coverage of BSJ read pairs and distribution of sequencing depth, to determine whether a candidate cirexon is continuously included in the circRNA. The sequencing depth is calculated based on recorded local alignment of sequencing reads. For each base within and outside a given circRNA, the sequencing depth may result from sequencing of circRNA, linear RNA or both, and the existence of a cirexon will cause remarkable variation of sequencing depth between inside and outside of its ends. CIRI-AS captures such variation by calculating the significance based on a Mann–Whitney *U*-test.

After filtering false candidates by detecting variation of sequencing depth, CIRI-AS will attempt to detect the truly existed cirexons inside false candidates (Supplementary Fig. 1B). Local alignments of non-BSJ read pairs mapping within the circRNA are analysed to retrieve the missing FSJs. A candidate of such FSJ should have both 5′- and 3′-splicing sites inside the false candidate and have no equivalent competitor that shares the same 5′ or 3′-splicing site with them. CIRI-AS will then check the distribution of sequencing depth inside the ‘new' cirexon candidates indicated by such splice junction once more as described above.

### Detection and Ψ value estimation of AS events in circRNAs

CIRI-AS compares relative positions of cirexons and FSJs that are supported by BSJ read pairs to detect AS in circRNAs. First, detected cirexons within a circRNA are traversed according to the corresponding FSJs, linking them to construct all possible routes between the circular junction donor and acceptor, which represent potential circular transcripts. Second, all cirexons in these routes are sorted based on their relative positions, which facilitates to make a comparison between these routes and to find candidates of AS event. Such candidates are then clustered into groups, which include alternatively spliced cirexon and their equivalents, and can be used to determine the type of AS events such as ES, A5SS, A3SS and IR. A skipped exon is detected when its two ends are within the donor and acceptor sites of a FSJ supported by BSJ read pairs. Two cirexons with alternative 5′/3′-splicing site are detected when they share one end with each other but have the other spliced differently and supported by BSJ read pairs. A retained intron is detected when BSJ read pairs can cover its both ends and the corresponding flanking cirexons.

To rule out potential interference from mRNA transcripts, the relative abundance of each AS event in circRNAs can be simply calculated based on counting the number of supporting BSJ read pairs. For example, FSJs splicing a cirexon in or out are the key FSJs of an ES-type event. Thus, the ratio of BSJ read counts supporting FSJs of each isoform can be calculated as an estimate of relative abundance of AS events. However, considering the BSJ reads are ‘anchored' at BSJ, the sampling of these reads is different from randomly sampled forward-spliced reads from linear transcripts. To eliminate the systemic bias caused by such biased sampling, CIRI-AS adopts a correction method based on the distribution of library insert lengths. CIRI-AS records long exons never alternatively spliced according to the annotation provided by users and calculates insert lengths of the read pairs mapping to the long exons to estimate the total distribution. For an AS event, CIRI-AS counts supporting BSJ read pairs in two strands and calculates corresponding insert lengths within a circle *K*. For each strand, supporting read count *c* is normalized by the sum of probabilities for the insert length in a circle *P(K)* and more than one circle P(K+J*i), where *J* and *i* denote the circumference of the isoform and number of circles, respectively. As a supporting read can be either the forward or the reverse read of a read pair, which corresponds to different insert lengths *K*, *c*_max_ is designated as the larger *c*-value of the two and corresponds to *K*_max_, whereas *c*_min_ is designated as the other. Thus, the normalized value *c'* of supporting read count *c* can be calculated as follows.

If *c*_max_ >>*c*_min_,


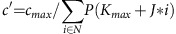


Else,


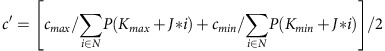


where *N* denotes non-negative integers.

The ratio of normalized read counts is calculated as the estimate of relative abundance.


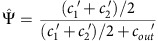


where *c*_1_′ and *c*_2_′ denote normalized count of BSJ read pairs split mapped to the 5′- and 3′-splicing sites of the exon, respectively, and *c*′_out_ denotes normalized count of BSJ read pairs split-mapped to both upstream and downstream constitute exons with the exon skipped. It has to be mentioned that only circRNAs with a single group of skipped cirexons (which may be alternatively spliced) can be estimated using the correction approach above. circRNAs with nested AS patterns, such as multiple independent skipped cirexons, have multiple routes and circumferences, and thus are not applicable to relative abundance correction. For these nested AS events, CIRI-AS only provides estimates of relative abundance without correction.

### Simulated data

CIRI simulator described previously^9^ was adapted for data simulation in this study. The total distribution of insert lengths is simulated by a single normal distribution or mixing two normal distributions in assigned percentages. Specifically, insert length (L) of a read pair, which obeys a distribution comprising two normal distributions ((*μ*_*1*_, *σ*_*1*_^2^) and (*μ*_*2*_, *σ*_*2*_^2^)) in the ratio of *p* to 1−*p*, is calculated using Box–Muller transform by generating three uniformly distributed random numbers *x*_1_, *x*_2_ and *x*_3_ independent to each other:

If *x*_1_ ≤*p*


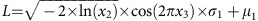


Else,


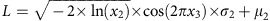


where


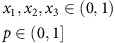


ES, a typical type of AS, can be simulated by CIRI simulator. CIRI simulator first simulates circRNAs with at least three fragments and randomly selects a cirexon from all cirexons within the simulated circRNA, except for the two cirexons at the end. Sequencing reads are then generated for the two circular transcripts that contains the spliced cirexon or not, according to an assigned Ψ value.

Different transcriptomic sequencing data were simulated using the adapted CIRI simulator. In detail, the whole-genome assembly of human (GRCh37) and the corresponding GTF annotation (Gencode version 19, downloaded from http://www.gencodegenes.org/releases/) were input as reference and annotation, respectively. Four distributions of insert lengths (*μ*=280 bp, *σ*=80 bp; *μ*_1_=280 bp, *σ*_1_=80 bp, *μ*_2_=370 bp, *σ*_2_=100 bp, *P*=0.9; *μ*=400 bp, *σ*=80 bp; *μ*_1_=550 bp, *σ*_1_=80 bp, *μ*_2_=280 bp, *σ*_2_=100 bp, *P*=0.85), ten sequencing depths (5-, 10-, 15-, 20-, 25-, 30-, 40-, 50-, 75- and 100-fold) and four read lengths (75, 100, 125 and 150 bp) were simulated for evaluation of CIRI-AS. To test the accuracy of Ψ estimation, circRNAs containing skipped exons with three Ψ values (0.25, 0.5 and 0.75) at four sequencing depths (25-, 50-, 75- and 100-fold) were simulated.

### Complementary DNA library preparation and sequencing

HeLa and HEK293 cells were grown in standard media and conditions. For each cell line, total RNA was isolated using TRIZOL and then was divided into six replicates. A RiboMinus kit (Invitrogen, Carlsbad, CA) was used to deplete ribosomal RNA in three replicates, two of which were further incubated at 37 °C and treated by 10 U μg^−1^ RNase R (Epicentre, Madison, WI). rRNA- and rRNA-/RNase R-treated samples were used as templates for cDNA libraries separately per TruSeq protocol (Illumina, San Diego, CA), whereas another replicate of total RNA was used for preparation of poly(A)-selected library per TruSeq v2 guide. The three libraries of HEK293 were then sequenced on the Illumina HiSeq 2500 platform of the Research Facility Center at Beijing Institutes of Life Science, CAS, with paired-end 150 bp (PE150) reads. The three cDNA libraries of HeLa cells were sequenced in the same platform with paired-end 100 bp (PE100) reads. A large insert library of HeLa cells was constructed using the same RNA sample but sequenced with paired-end 250 bp (PE250) reads for a validation of cirexon predictions by CIRI-AS.

### Public data

rRNA- (SRR444655 and SRR444975) and rRNA-/RNase R-treated (SRR445016 and SRR444974) RNA-seq data for Hs68 generated in a previous study^4^ were downloaded from NCBI SRA database. RNA-seq data sets for mouse immune dendritic cells were downloaded from the NCBI website http://www.ncbi.nlm.nih.gov/geo/query/acc.cgi?acc=GSE56977. Nucleus/cytosol long poly(A)− RNA data for all seven available cell lines (GM12878, H1-hESC, HUVEC, HeLa-S3, HepG2, K562 and NHEK) as well as the corresponding long poly(A)+ RNA data in ENCODE were downloaded from the UCSC website http://genome.ucsc.edu/cgi-bin/hgFileUi?db=hg19&g=wgEncodeCshlLongRnaSeq. All 103 *D. melanogaster* total RNA data sets generated in a previous study^24^ were downloaded from NCBI SRA database according to Table S1 of the report.

### Cirexon detection and rarefaction analysis

For human cell line samples, genome assembly of GRCh37 and Gencode version 19 annotation were used as genome reference and annotation, respectively. For mouse samples, genome GRCm38 and version GRCm38.76 GTF-formatted annotation were used as genome reference and annotation, respectively. For *D. melanogaster* samples, BDGP6 genome and version 6.81 GTF-formatted annotation were used as genome reference and annotation, respectively. Read alignment of each simulated or real data set was performed by BWA-MEM with ‘-T 19' and default parameters. The SAM alignment, genome reference and GTF-formatted annotation mentioned above were used as input of CIRI for circRNA detection with default parameters. The circRNA list output by CIRI and the corresponding SAM alignment, genome reference and GTF annotation mentioned above were used as input of CIRI-AS for cirexon and AS detection with default parameters. For simulated data, a customized script was used for comparison of cirexons detected and simulated.

rRNA-/RNase R-treated RNA-seq data of fibroblast Hs68 (SRR445016) mentioned above was used for a rarefaction analysis of cirexons and the corresponding BSJs. The rarefaction analysis was based on prediction results of CIRI and CIRI-AS on ten subsets of the data and repeated for three times. In each repeat, each read in the data set was put into one of the ten subsets by a customized script using a random number. Each of the ten subsets was then added to an accumulated set that was processed by CIRI and CIRI-AS. The rarefaction curves were generated using LOESS regression fitting based on prediction counts in the three repeats.

### Validation of detected cirexon using long-read sequencing

The paired-end 250 bp sequencing reads was first merged into long reads with an average length of 347.5 bp using FLASH^38^ with ‘-M 250' and default parameters. The long reads were then searched against themselves using BLASTN. A confident hit between two ends of the same long read may indicate a circRNA that was sequenced more than one circle within the long read. Such circRNA candidates were clustered according to their start and end positions, and compared with those detected by CIRI. The long reads supporting the shared circRNAs of the two approaches were then trimmed to obtain the whole sequence of circRNAs. Comparison between the circRNA sequence and cirexons detected by CIRI-AS was performed. A gap was used as a query against the genome reference by BLASTN to ascertain whether it was a cirexon missed by CIRI-AS.

### Comparison of skipped cirexons between circRNA and mRNA

The cirexons that are detected as skipped cirexons by CIRI-AS both in HeLa and HEK293 rRNA-/RNase R-treated samples were recorded with their Ψ values. The Ψ values of the corresponding exons were then calculated from poly(A)+ samples of HeLa and HEK293 cells using the same criteria as CIRI-AS. For exons that were constitute or absent in mRNAs, their Ψ values were recorded as 1 or 0, respectively. We performed statistical tests based on a β-binomial model, adapted from a previous approach (CircTest)^15^, except that we replaced the observed read counts from linear or circular isoforms with those from circular transcript splicing in or out of a detected exon. For each pair of the four groups of skipped exons, Euclidean distances based on the Ψ values were calculated and used for clustering.

### Experimental validation

Outward-facing primer sets were designed for cirexons detected by CIRI-AS. cDNA of poly(A)+, total RNA and rRNA-/RNase R-treated samples of HeLa cells, as well as DNA samples, were used as templates of PCR. All PCR reactions were performed using 35 cycles for the three cDNA samples and genomic DNA. The PCR products were then cloned and their sequences were determined by Sanger sequencing, to validate both circularity and cirexons.

To verify the existence of predicted circRNA isoforms and their relative abundances, equal amount of total RNA isolated from HeLa cells was converted to cDNA using random hexamers primers within the SuperScript III First-Strand Synthesis System (Life Technologies) directly or after RNase R (Epicentre) treatment. The resulted cDNA was used as templates and real-time qPCR was performed using primer pairs designed for nine circRNA-specific and two negative controls (GAPDH and β-actin) with SYBR FAST qPCR Kits (Kapa Biosystems). The reaction volume was 20 μl, which contained 1 μl of serial diluted cDNA, 10 μl of SYBR Master Mix, 0.5 μl each of forward and reverse primers, and 8 μl of water. Thermal cycling was carried out on Agilent Mx3000P Systems (Agilent Technologies) using the following conditions: 95 °C for 3 min and followed by 40 cycles of 95 °C for 15 s and 60 °C for 1 min. Fluorescent signals were detected at the step of annealing/extension (60 °C).

### Binding site density of regulatory factors in exons

Skipped/constitutive mRNA exons and skipped/constitutive cirexons in the HeLa data set were detected using Cufflinks v2.2 (ref. 33) and CIRI-AS, respectively. Annotated exons were randomly selected according to GTF-formatted Gencode annotation using a custom script. The binding sites for 21 human splicing factors and 95 RNA-binding proteins were then predicted for each exon and its adjacent intronic/intergenic sequences with two folds of exon lengths using online tools SFmap^29^ and RBPmap^30^, respectively. The binding density was then calculated as the percentage of binding sites in the whole length of the sequence for prediction. For each subset of exons, such binding densities of all exons with at least one binding site was calculated and the mean of their binding density was shown in the heatmap after *Z*-score normalization. The statistical significances of comparison between skipped cirexons and skipped mRNA exons, as well as between skipped exons and the corresponding constitutive exons, were calculated using Mann–Whitney *U*-tests with an FDR correction.

### Comparison of AS cirexons between the nucleus and cytosol

Long poly(A)+ RNA data in ENCODE for the seven cell lines mentioned above were processed by TopHat v2.0.9 (ref. 39) using default parameters against the whole genome assembly of human (GRCh37) with the corresponding GTF annotation. AS events and corresponding expression were detected using AltAnalyze^40^ with default parameters for RNA-seq reads. Corresponding long poly(A)-/rRNA-RNA data were processed by CIRI-AS as mentioned above. The detection results of both CIRI-AS and AltAnalyze were processed using a customized script to produce Fig. 4a,b.

### Analyses of circRNA AS events in *D. melanogaster* samples

Cirexons alternatively spliced in at least one of the *D. melanogaster* samples were extracted with their relative abundances from outputs of CIRI-AS. Heatmap for the relative abundances was produced using heatmap.2 function in ‘gplots' package of R and the clustering was performed using default settings in the same package. Gene Ontology enrichment analysis for parental genes of alternatively spliced circRNAs in adult heads and laval/pupal CNS was performed in clusterProfiler package^41^ version 2.2.4 of R using default settings, with the parental genes of all alternatively spliced circRNAs in *D. melanogaster* samples as the background. Principal components analysis was performed in imDEV^42^ using default settings.

### Data availability

The RNA-seq data of HeLa and HEK293 cell line generated in this study have been deposited in the BioProject database of Genbank under the accession number PRJNA266072. Specifically, sequencing data of rRNA-, rRNA-/RNase R-treated and poly(A)-selected samples of HeLa cell were submitted to NCBI SRA under the accession numbers SRR3476958, SRR3476956 and SRR3479116, respectively. Sequencing data of rRNA-, rRNA-/RNase R-treated and poly(A)-selected samples of HEK293 cell were submitted to NCBI SRA under the accession numbers SRR3479243, SRR3479244 and SRR3479143, respectively. Details about data generated in previous studies and analysed in this study were included in Supplementary Table 1 and the Methods section. The authors declare that all other data supporting the findings of this study are available within the article and its Supplementary Information files.

## Additional information

**Accession codes:** The RNA-seq data of HeLa and HEK293 cell line generated in this study have been deposited in the BioProject database of Genbank under the accession number PRJNA266072. Specifically, sequencing data of rRNA-, rRNA-/RNase R-treated and poly(A)-selected samples of HeLa cell were submitted to NCBI SRA under the accession numbers SRR3476958, SRR3476956 and SRR3479116, respectively. Sequencing data of rRNA-, rRNA-/RNase R-treated and poly(A)-selected samples of HEK293 cell were submitted to NCBI SRA under the accession numbers SRR3479243, SRR3479244 and SRR3479143, respectively.

**How to cite this article**: Gao, Y. *et al.* Comprehensive identification of internal structure and alternative splicing events in circular RNAs. *Nat. Commun.* 7:12060 doi: 10.1038/ncomms12060 (2016).

## Supplementary Material

Supplementary Information Supplementary Figures 1-26 and Supplementary Tables 1-10
