# Supplementary material for: Comprehensive identification of internal structure and alternative splicing events in circular RNAs
Source: Nat Commun. 2016 Jun 28;7:12060. doi: 10.1038/ncomms12060 (PMC4931246; doi:10.1038/ncomms12060)
Supplement: Supplementary Information — Supplementary Figures 1-26 and Supplementary Tables 1-10 [file ncomms12060-s1.pdf]

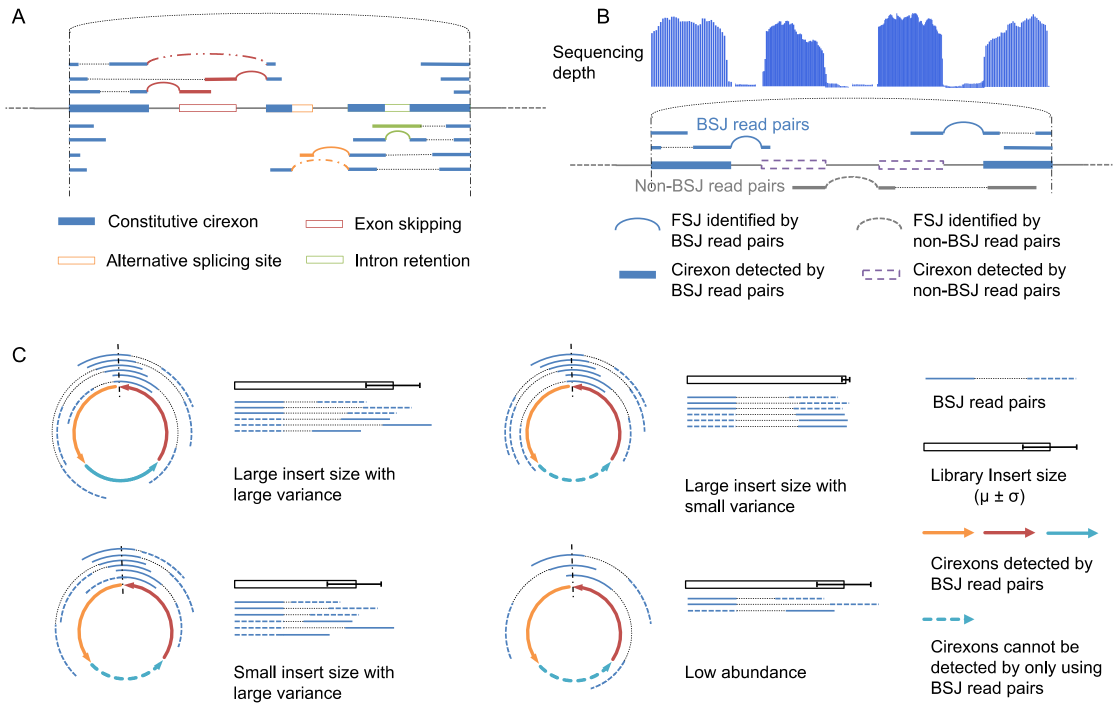

Supplementary Figure 1. Alternative splicing and cirexon detection in CIRI-AS. (A) Split-alignment of BSJ read pairs in detecting exon skipping, alternative 5'/3' splicing site and intron retention events. (B) Sequencing depth variation and non-BSJ read pairs are used to detect FSJs and cirexons missed by BSJ-read pairs. (C) Impact of library insert sizes and variances as well as sequencing depth on FSJ detection by BSJ read pairs.

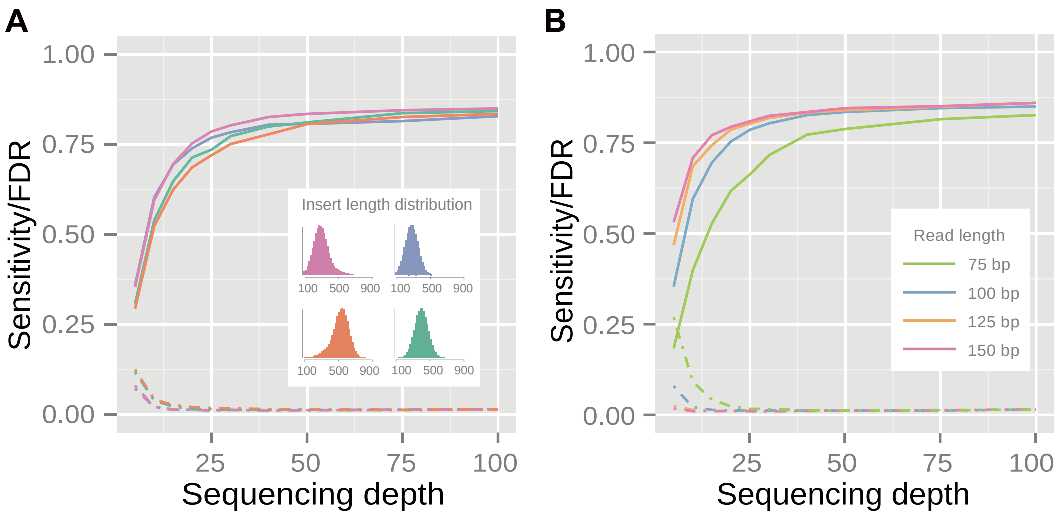

Supplementary Figure 2. **Performances of CIRI-AS for cirexon detection on simulated data sets.** (A) Sensitivity and FDR for cirexon detection with varying sequencing depths in simulated data sets (PE100 bp) for four distributions of insert lengths. (B) Sensitivity and FDR for cirexon detection with varying sequencing depths in simulated data sets (read length distribution parameter: μ_1=280 bp, σ_1=80 bp, μ_2=370 bp, σ_2=100 bp, p=0.9) for four sequencing read lengths.

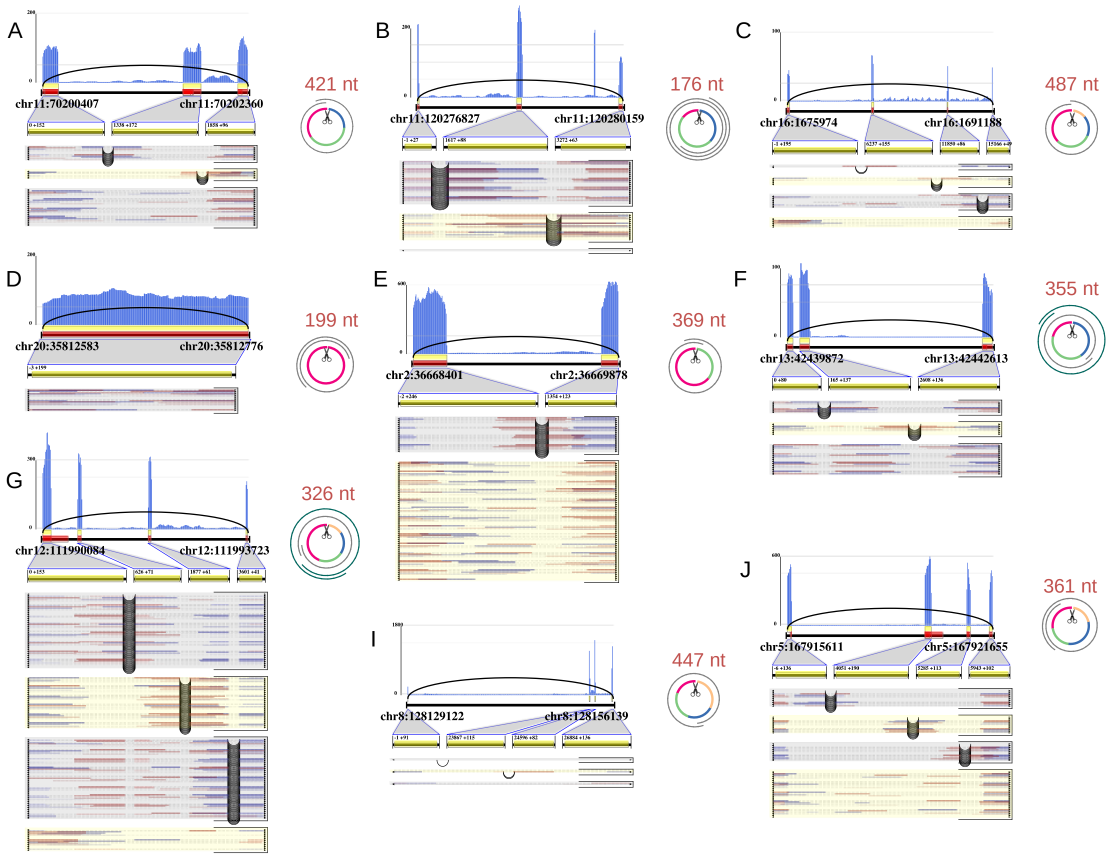

Supplementary Figure 3. **Examples of validated cirexons detected by CIRI-AS using long-read data.** Detected cirexons by CIRI-AS were shown on left and the corresponding components identified in long-read data were shown on right.

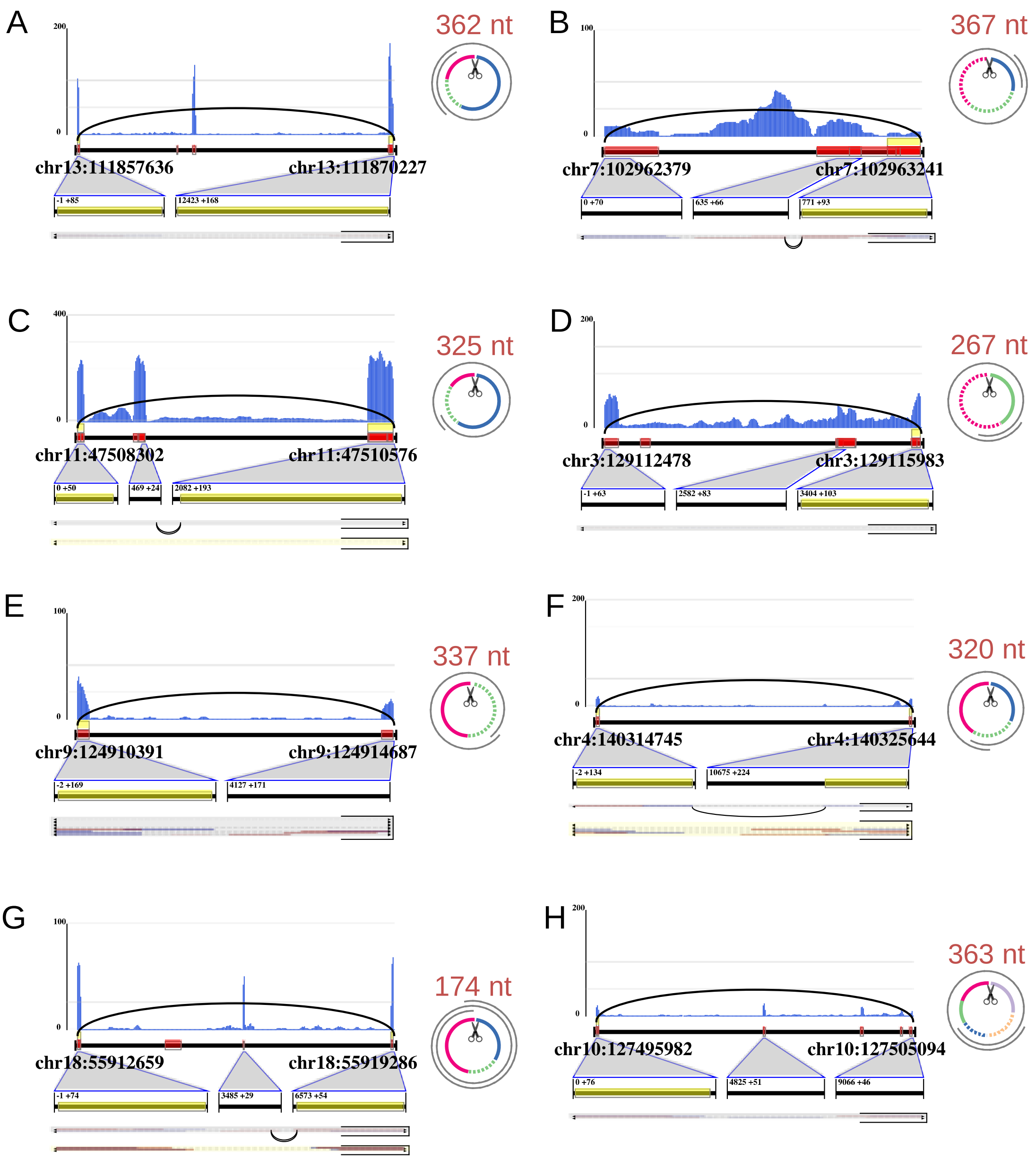

Supplementary Figure 4. **Examples of cirexons identified using long-read data but not detected by CIRI-AS using 2 × 100 bp paired reads.** Detected cirexons by CIRI-AS were shown on left and the corresponding components identified in long-read data were shown on right. Inner colored solid-line curves indicate the cirexons detected by CIRI-AS, whereas the dashed-line curves represent the cirexons missed by CIRI-AS.

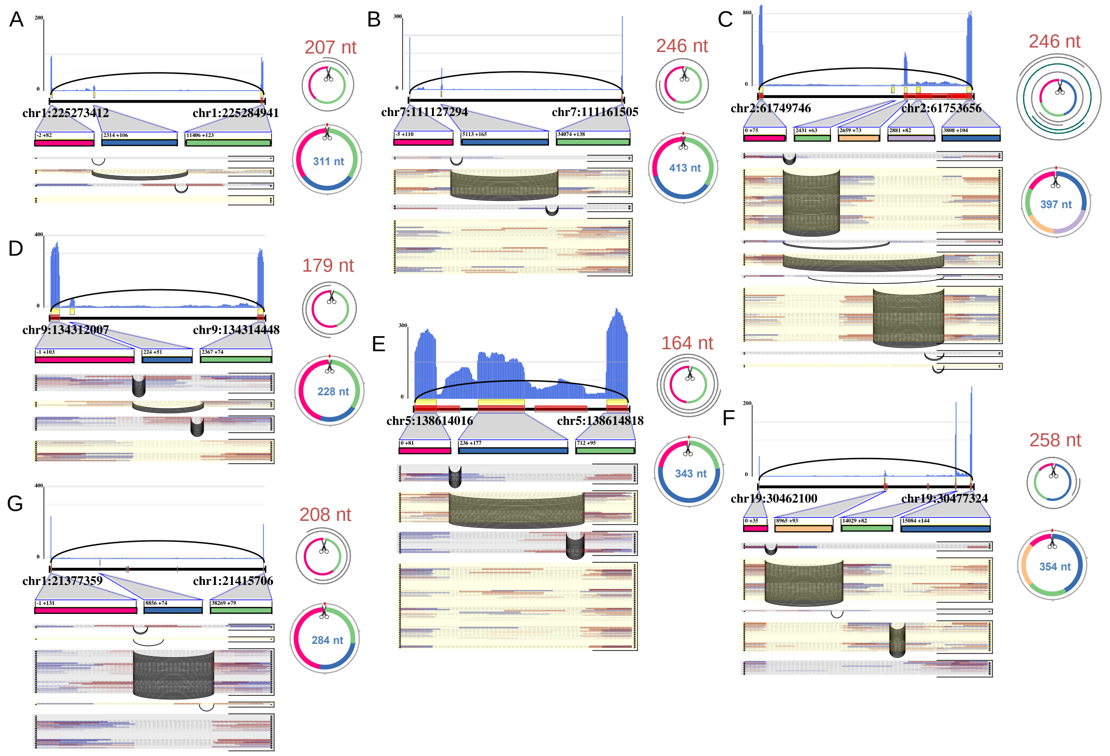

Supplementary Figure 5. Application of CIRI-AS on 2 × 100 bp paired reads can detect more isoforms than low-coverage long-read based identification. Detected cirexons by CIRI-AS were shown on left and the corresponding components identified in long-read data were shown on right. The internal compositions of alternatively splice transcripts not identified in long-read data were shown in the left bottom.

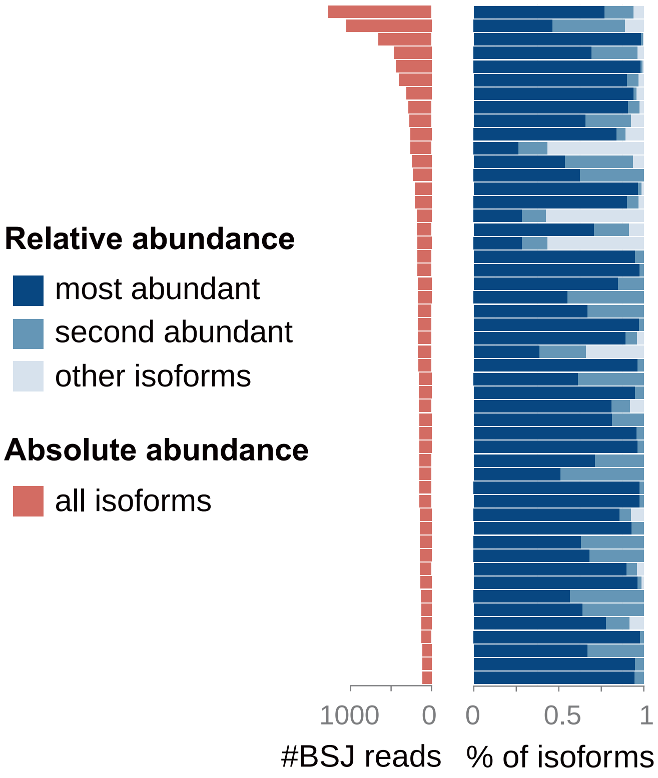

Supplementary Figure 6. The absolute abundances of top 50 most abundant alternatively spliced circRNAs as well as relative abundances of their AS transcripts detected in RNase R+ sample of HeLa cells.

A

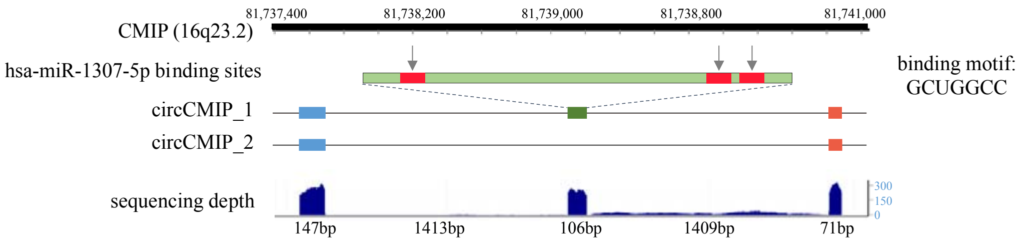

B

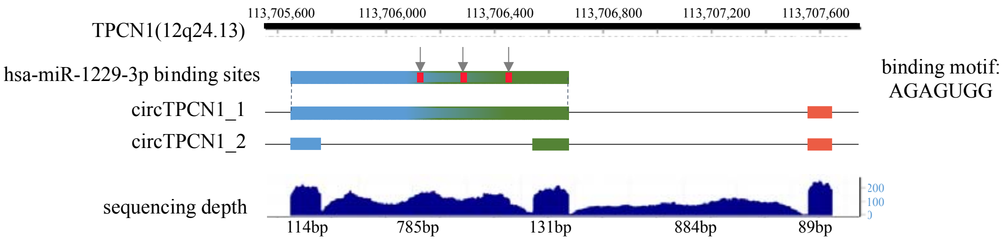

Supplementary Figure 7. Examples of the density of miRNA binding sites within circRNAs altered by AS events.

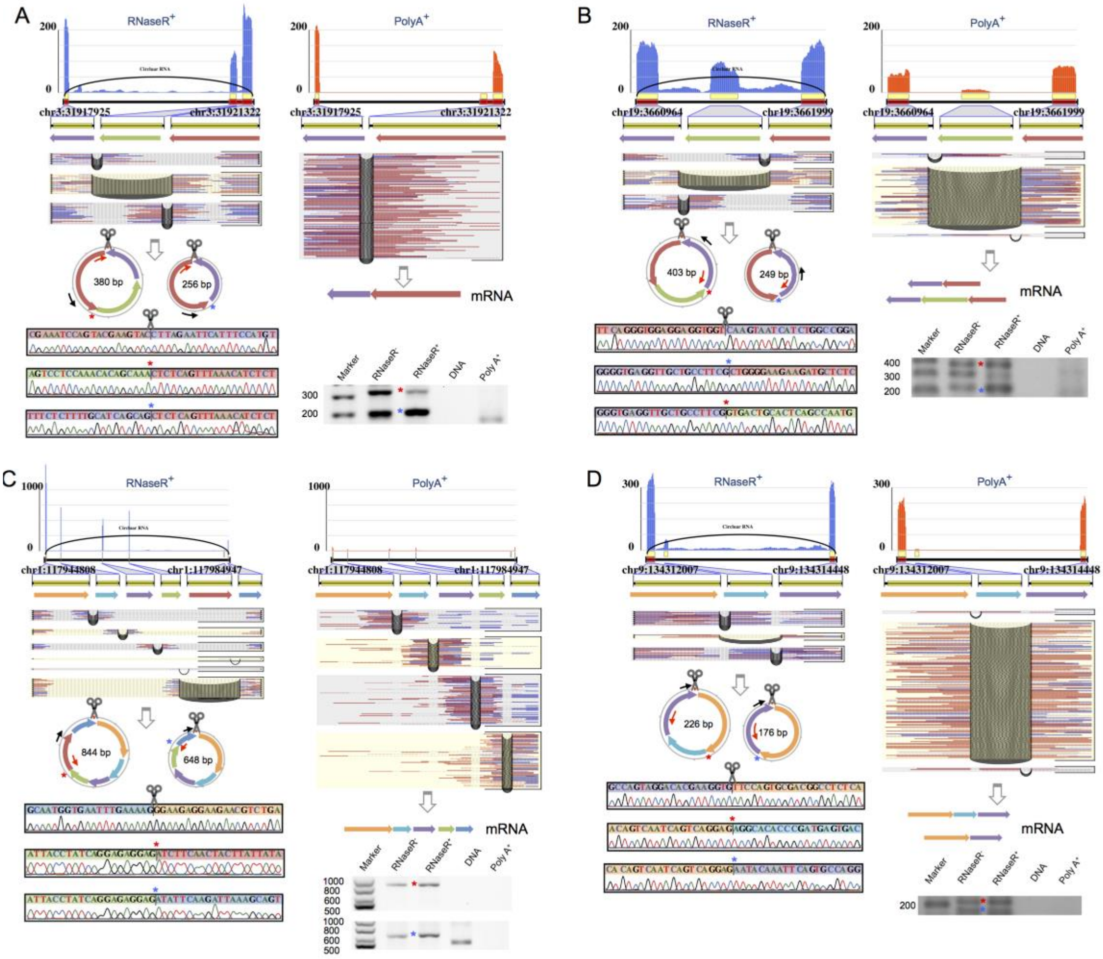

Supplementary Figure 8. **Circular transcripts with exon skipping validated by RT-PCR and Sanger sequencing in HeLa cells.** For the FSJs in transcripts with exon skipping, only related BSJ read pairs were shown. Reads in poly(A) selected sample of HeLa cells that indicate the corresponding splice junctions were shown in the right column.

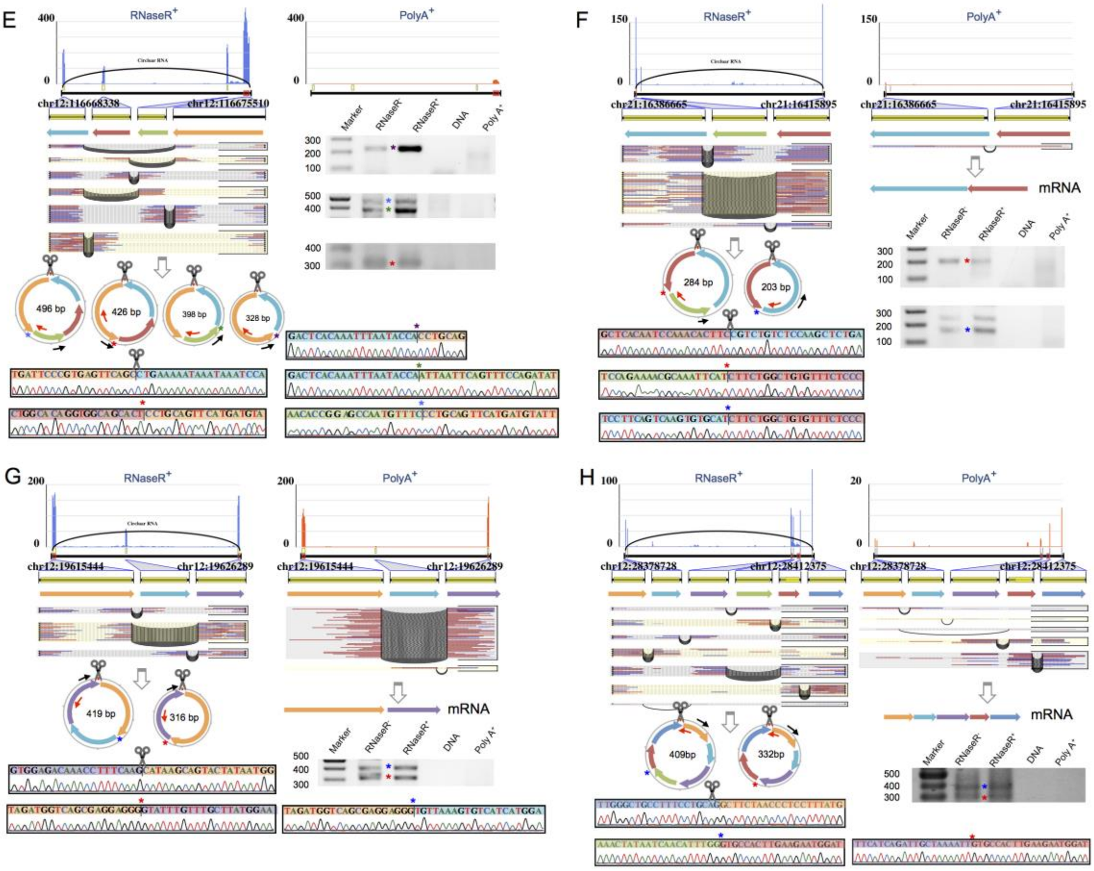

Supplementary Figure 8. (continued)

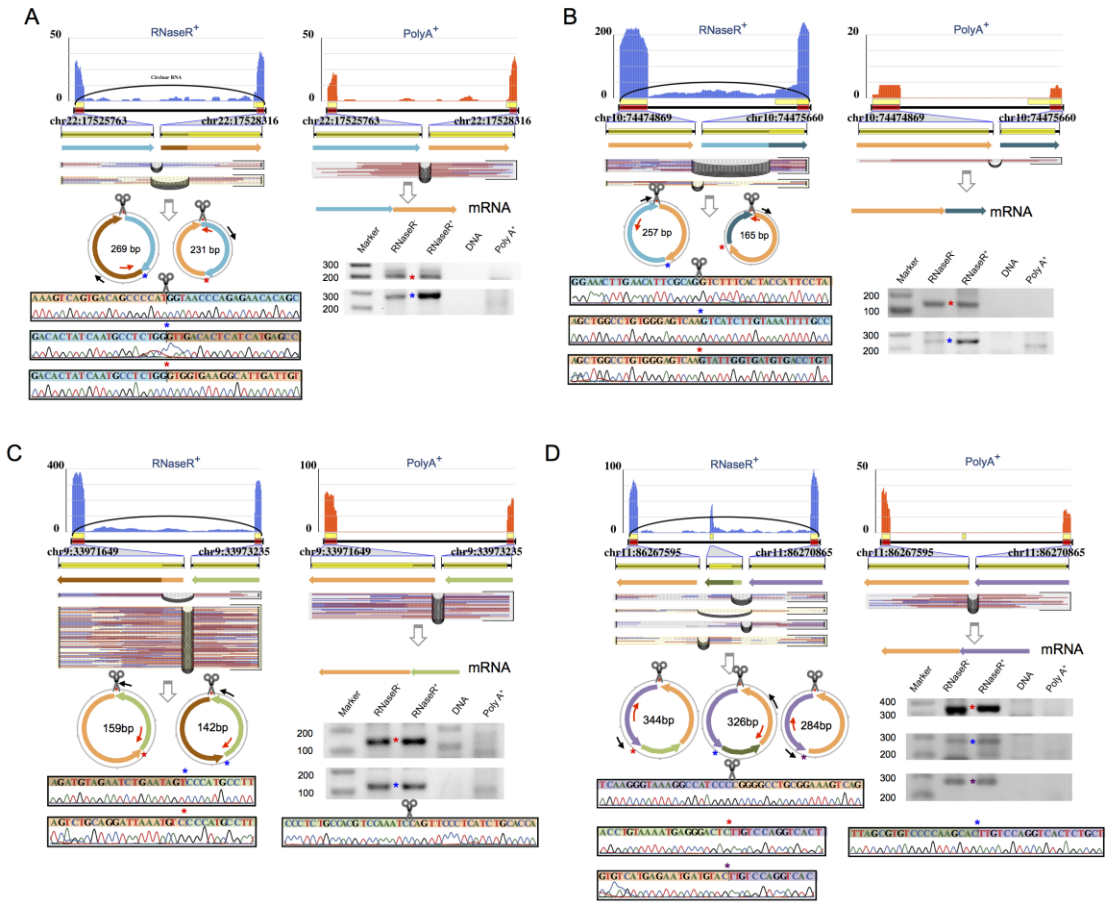

Supplementary Figure 9. Circular transcripts with alternative 3' splicing site validated by RT-PCR and Sanger sequencing in HeLa cells. For the FSJs in transcripts with alternative 3' splicing site, only related BSJ read pairs were shown. Reads in poly(A) selected sample of HeLa cells that indicate the corresponding splice junctions were shown in the right column.

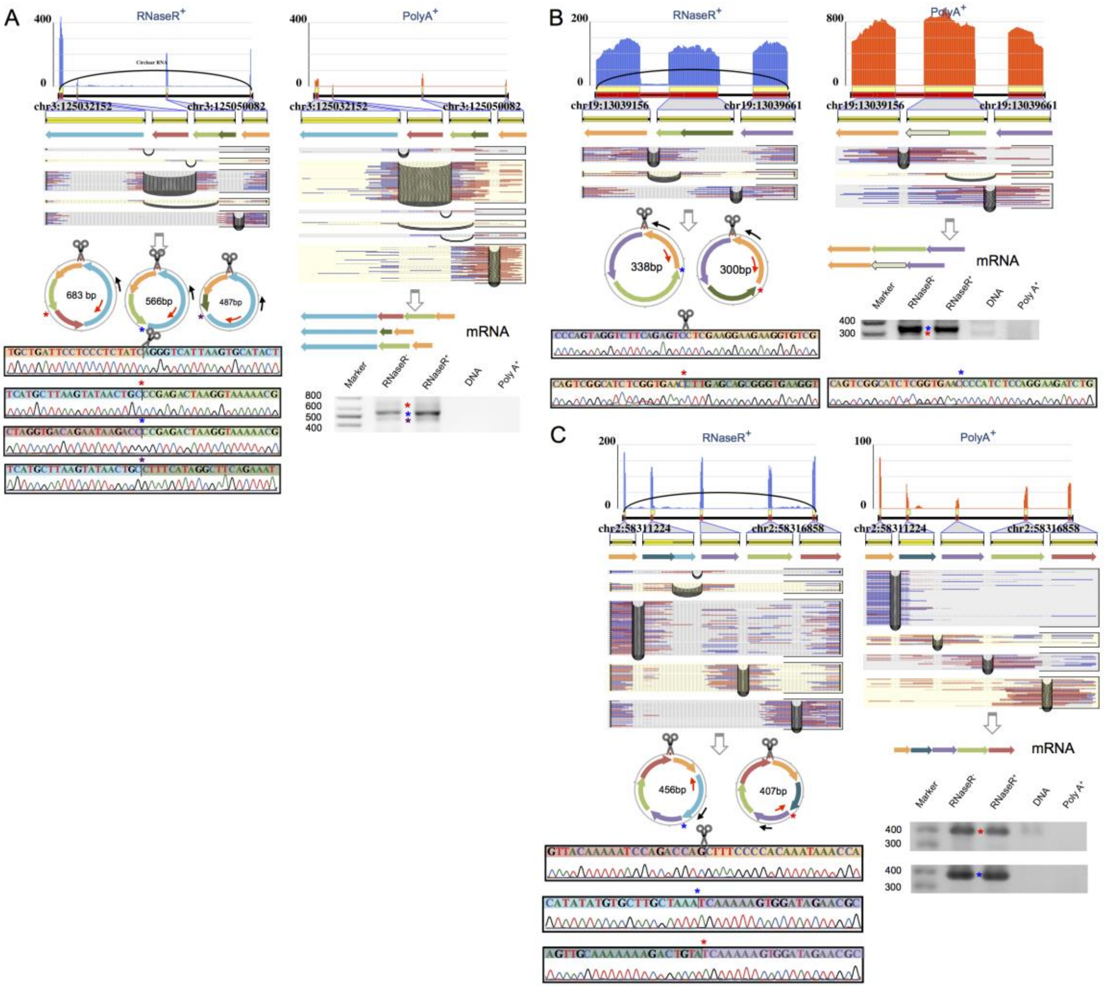

Supplementary Figure 10. **Circular transcripts with alternative 5' splicing site validated by RT-PCR and Sanger sequencing in HeLa cells.** For the FSJs in transcripts with alternative 5' splicing site, only related BSJ read pairs were shown. Reads in poly(A) selected sample of HeLa cells that indicate the corresponding splice junctions were shown in the right column.

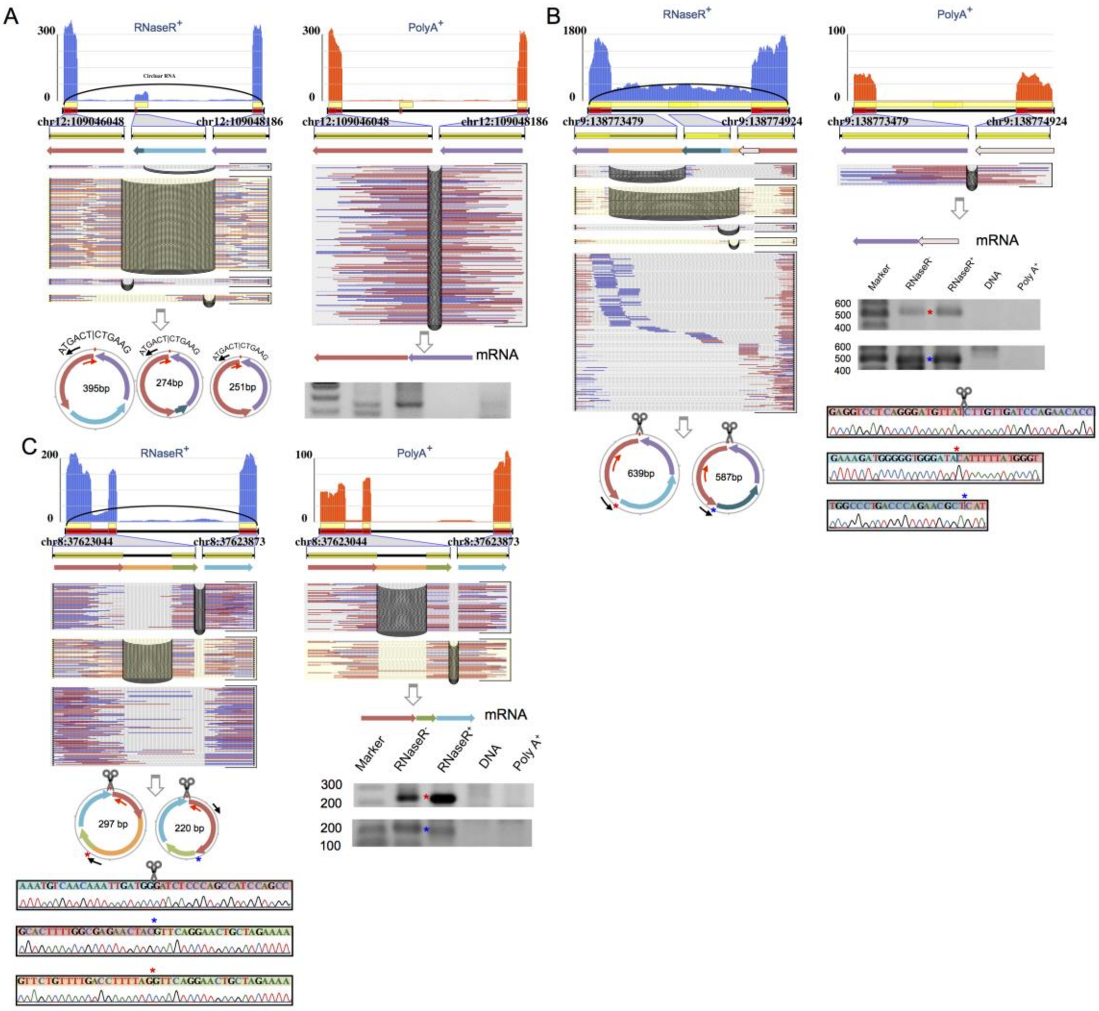

Supplementary Figure 11. Circular transcripts with intron retention validated by RT-PCR and Sanger sequencing in HeLa cells. For the FSJs and retained introns in the transcripts, only related BSJ read pairs were shown. Reads in poly(A) selected sample of HeLa cells that indicate the corresponding splice junctions were shown in the right column.

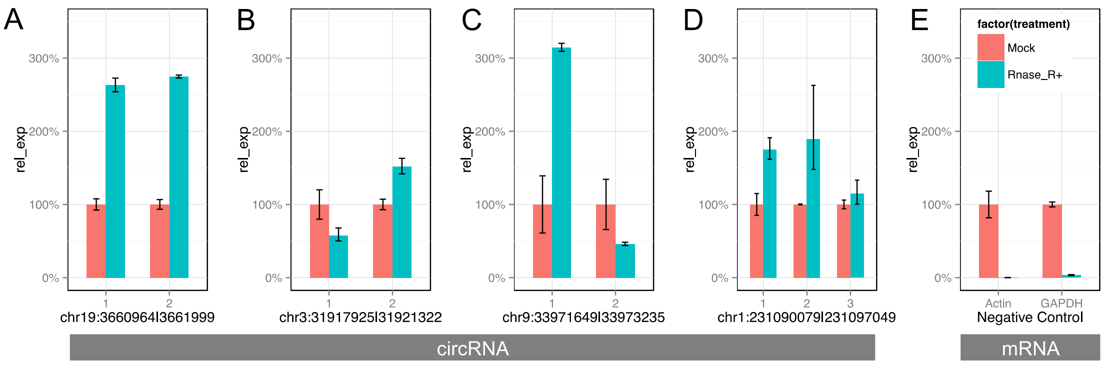

Supplementary Figure 12. **Resistence of circular AS isoforms to RNase R quantified by qRT-PCR.** (A-D) Nine circular AS isoforms of four circRNA loci (n = 3; mean ± SE). (E) Linear control of GAPDH and Actin (n = 3; mean ± SE). Red bars represent total RNA samples, whereas blue bars represent RNase R-treated RNA samples.

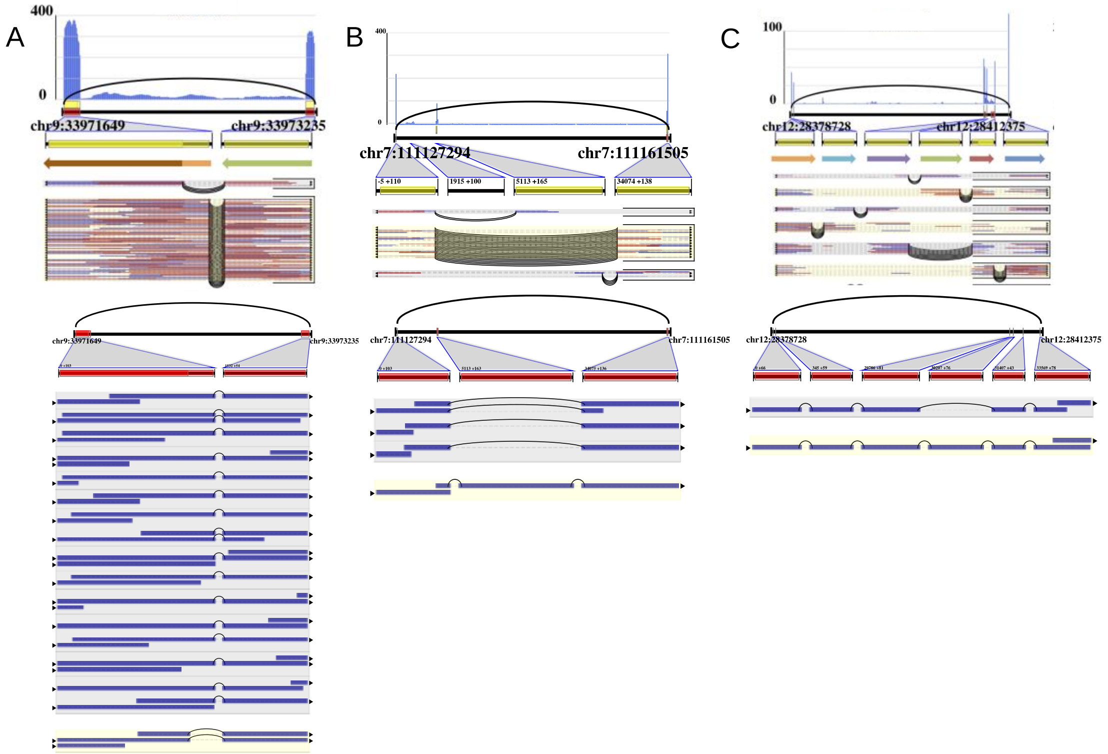

Supplementary Figure 13. Three examples of circular AS isoforms detected by both CIRI-AS (upper panel) and long-read sequencing (lower panel). In the lower panel, blue lines indicate the long reads that can cover the full length of circRNAs. Curved lines indicate the splicing junctions. Black triangles indicate BSJs located in the long reads.

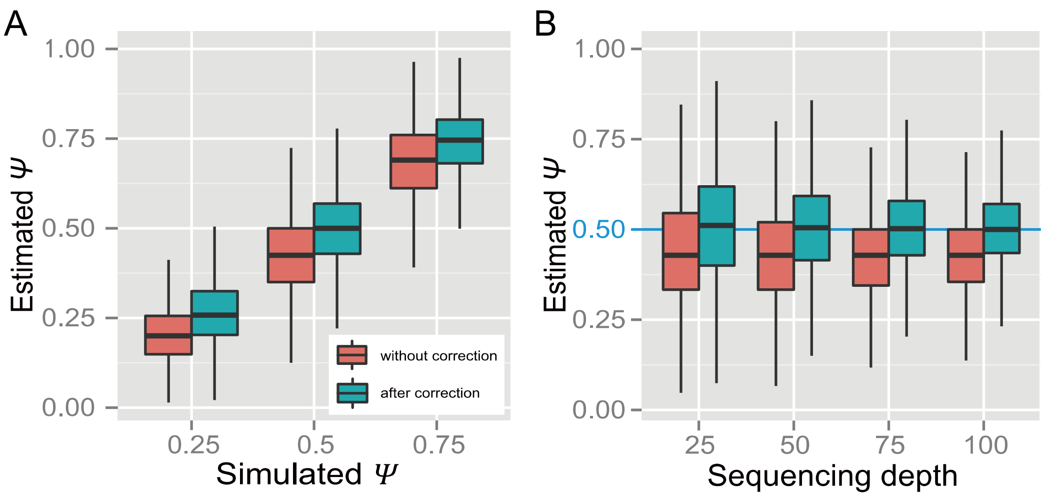

Supplementary Figure 14. **Performances of CIRI-AS for relative abundance estimation on simulated data sets.** (A) Estimated Ψ values without correction or after correction in CIRI-AS for exon skipping within simulated circRNAs with three simulated Ψ values. (B) Estimated Ψ values without correction or after correction in CIRI-AS for exon skipping within simulated circRNAs with four sequencing depths.

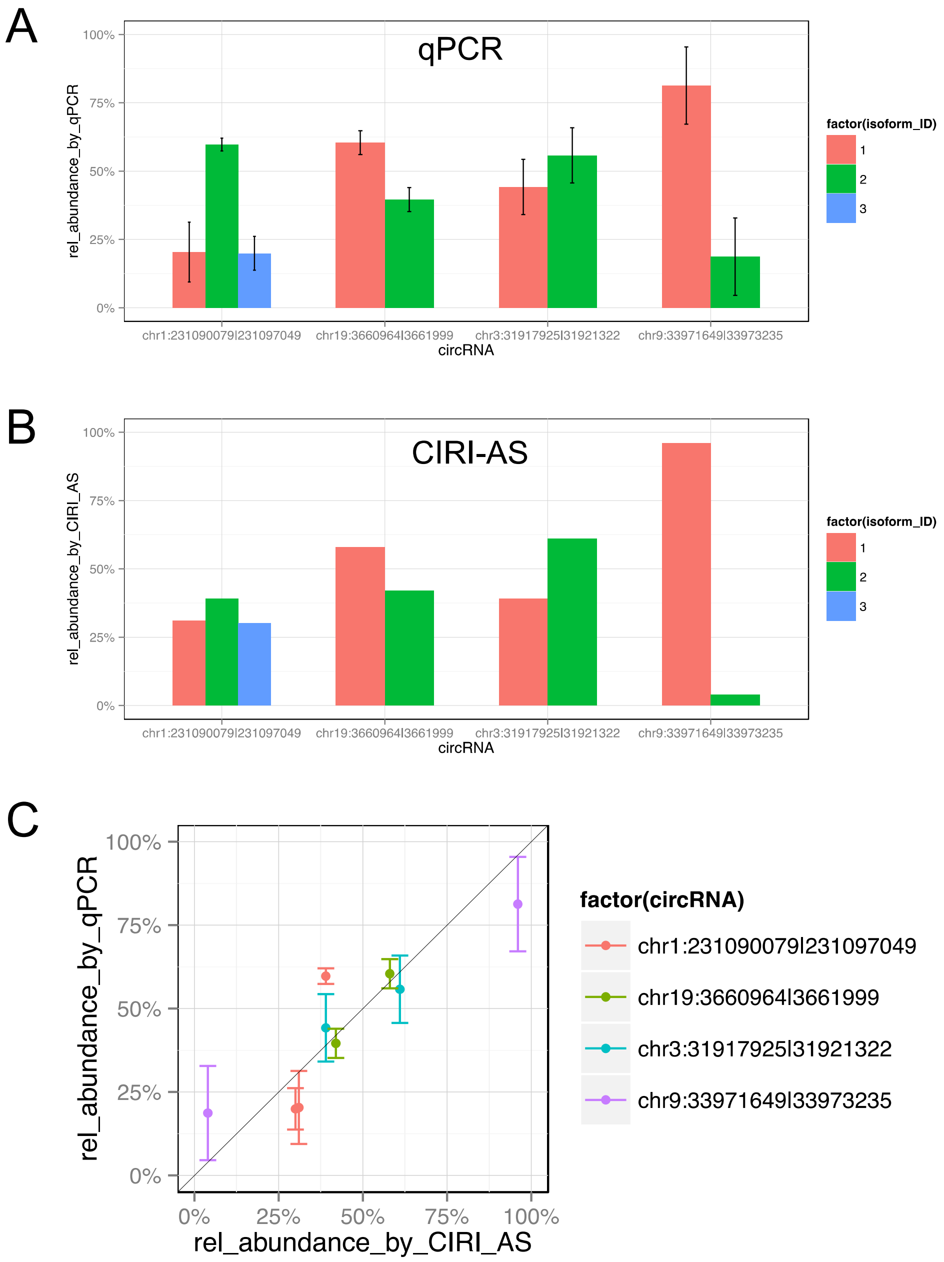

Supplementary Figure 15. Relative abundance of nine AS isoforms in four circRNA loci estimated by qRT-PCR and CIRI-AS. (A) Relative abundance of nine AS isoforms in four circRNA loci estimated by qRT-PCR (n = 4; mean ± SE); (B) Relative abundance of nine AS isoforms in four circRNA loci estimated by CIRI-AS; (C) Comparison of relative abundances estimated by CIRI-AS and qRT-PCR (n = 4; mean ± SE).

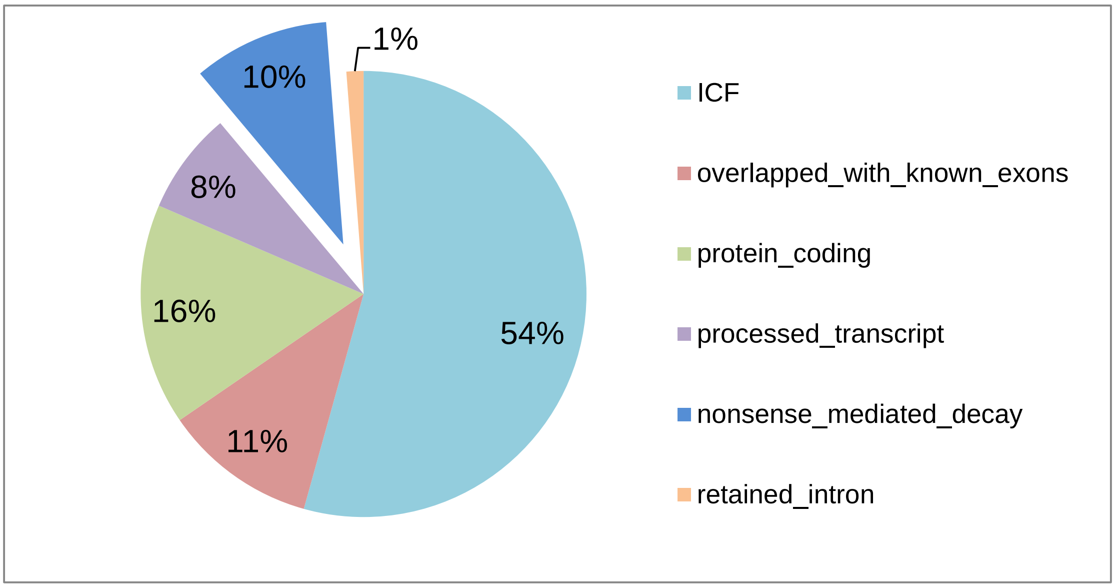

Supplementary Figure 16. Compositions of cirexons with higher relative abundance in circRNAs compared to mRNAs in HeLa and HEK293. Cirexon classification is based on the Gencode annotation.

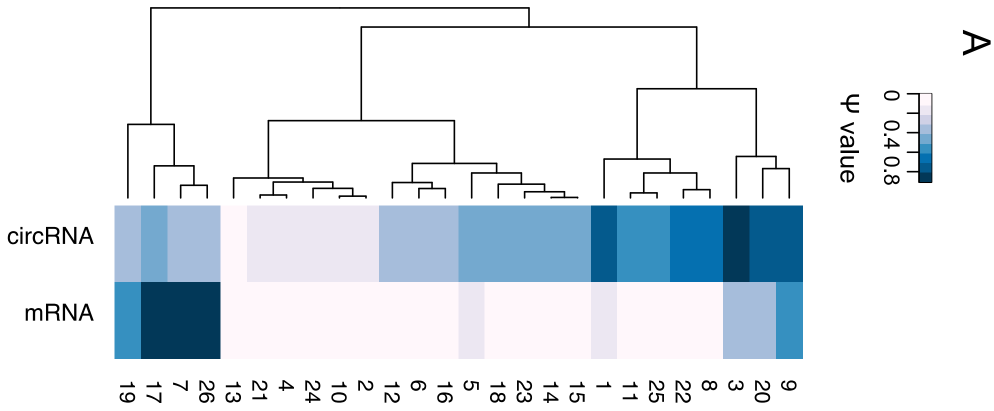

B

ID	circRNA	#BSJ	Gene	strand	cirexons	P_value
1	4:9635901|9639347	76	Asph	-	9639198:9639347,9637659:9637703,9635901:9635969,	**0.02028062**
2	6:47576536|47577667	52	Ezh2	-	47577544:47577667,47576536:47576664,47576536:47577667,47577276:47577391,	0.31250106
3	4:9583812|9610938	47	Asph	-	9610843:9610938,9607797:9607871,9604516:9604629,9602469:9602501,9601307:9601363,9598733:9598780,9598269:9598301,9595377:9595415,9594663:9594698,9583812:9583856,	0.33521847
4	4:9583812|9610938	47	Asph	-	9610843:9610938,9607797:9607871,9604516:9604629,9602469:9602501,9601307:9601363,9598733:9598780,9598269:9598301,9595377:9595415,9594663:9594698,9583812:9583856,	0.2310801
5	11:22053432|22068506	27	Ehbp1	-	22068359:22068506,22062780:22062887,22059137:22059261,22056783:22056921,22056530:22056627,22053432:22053555,	**0.05380791**
6	11:22053432|22068506	27	Ehbp1	-	22068359:22068506,22062780:22062887,22059137:22059261,22056783:22056921,22056530:22056627,22053432:22053555,	0.88502238
7	7:90189150|90197020	23	Picalm	+	90189150:90189257,90191141:90191272,90191156:90191272,90191359:90191389,90191654:90191753,90194273:90194296,90195655:90195714,90196916:90197020,	0.43224364
8	4:16151917|16158430	22	Ripk2	-	16158277:16158430,16157572:16157648,16155035:16155190,16151917:16152074,	0.55319232
9	1:39555478|39565332	21	Rnf149	-	39565082:39565332,39564048:39564116,39560693:39560775,39558315:39558411,39555478:39555670,	0.2930278
10	4:53127597|53133015	16	Abca1	-	53132922:53133015,53131589:53131669,53128721:53128862,53127597:53127715,	0.33521847
11	12:116317830|116324210	15	Esyt2	+	116317830:116317871,116318804:116318938,116319280:116319361,116321127:116321203,116323840:116323912,116324121:116324210,	0.10877534
12	1:160700712|160702460	14	Rabgap1l	-	160702358:160702460,160701691:160701796,160700712:160700878,	**0.07927107**
13	14:45616530|45629753	14	Ddhd1	-	45629580:45629753,45628854:45628874,45620712:45620840,45619269:45619416,45616530:45616636,	0.88502238
14	2:70770004|70786995	13	Tlk1	-	70786877:70786995,70784716:70784810,70784726:70784810,70770004:70770075,	0.14980362
15	1:139053346|139062971	13	Dennd1b	+	139053346:139053415,139054541:139054621,139059067:139059126,139060275:139060328,139062861:139062971,	0.37040131
16	17:81428182|81445593	10	Slc8a1	-	81445487:81445593,81444869:81444972,81442203:81442223,81428182:81428281,	0.26465869
17	1:172173943|172187460	10	Dcaf8	+	172173943:172174083,172175483:172175577,172175483:172175610,172179907:172180017,172186296:172186368,172186855:172186912,172187353:172187460,	**0.06300302**
18	12:86260577|86269061	10	Gpatch2l	+	86260577:86260753,86265182:86265249,86267144:86267183,86267240:86267294,86268976:86269061,	**0.07927107**
19	12:86260577|86269061	10	Gpatch2l	+	86260577:86260753,86265182:86265249,86267144:86267183,86267240:86267294,86268976:86269061,	**0.09158341**
20	4:9594663|9610938	10	Asph	-	9610843:9610938,9607797:9607871,9604516:9604629,9602469:9602501,9601307:9601363,9598733:9598780,9598269:9598301,9595377:9595415,9594663:9594698,	0.10877534
21	4:9594663|9610938	10	Asph	-	9610843:9610938,9607797:9607871,9604516:9604629,9602469:9602501,9601307:9601363,9598733:9598780,9598269:9598301,9595377:9595415,9594663:9594698,	0.37040131
22	3:60595594|60603894	7	Mbnl1	+	60595594:60595764,60596719:60596829,60603694:60603894,	**0.06394479**
23	6:134066307|134137417	5	Etv6	+	134066307:134066465,134115220:134115351,134137285:134137417,	0.52235464
24	8:78515586|78578757	5	Slc10a7	+	78515586:78515668,78525162:78525298,78531083:78531158,78554931:78555021,78578719:78578757,	**0.06394479**
25	1:177102962|177109738	4	Akt3	-	177109627:177109738,177109419:177109478,177102962:177103106,	**0.05039189**
26	13:45795509|45956575	3	Atxn1	-	45956455:45956575,45852328:45852456,45795509:45795637,	**0.05039189**

Supplementary Figure 17. Exon skipping events detected in mouse immune dendritic cells with 10 min labeling pulse of 4-thiouridine. (A) Comparison of Ψ value for exon skipping events between circRNA and mRNA. (B) Information about skipped cirexons including read counts, parent gene names of circRNAs, position of cirexons as well as P values after correction for multiple testing (corrected P values <0.10 are shown in bold). Skipped cirexons classified as ICFs are shown in red, and other skipped cirexons in blue.

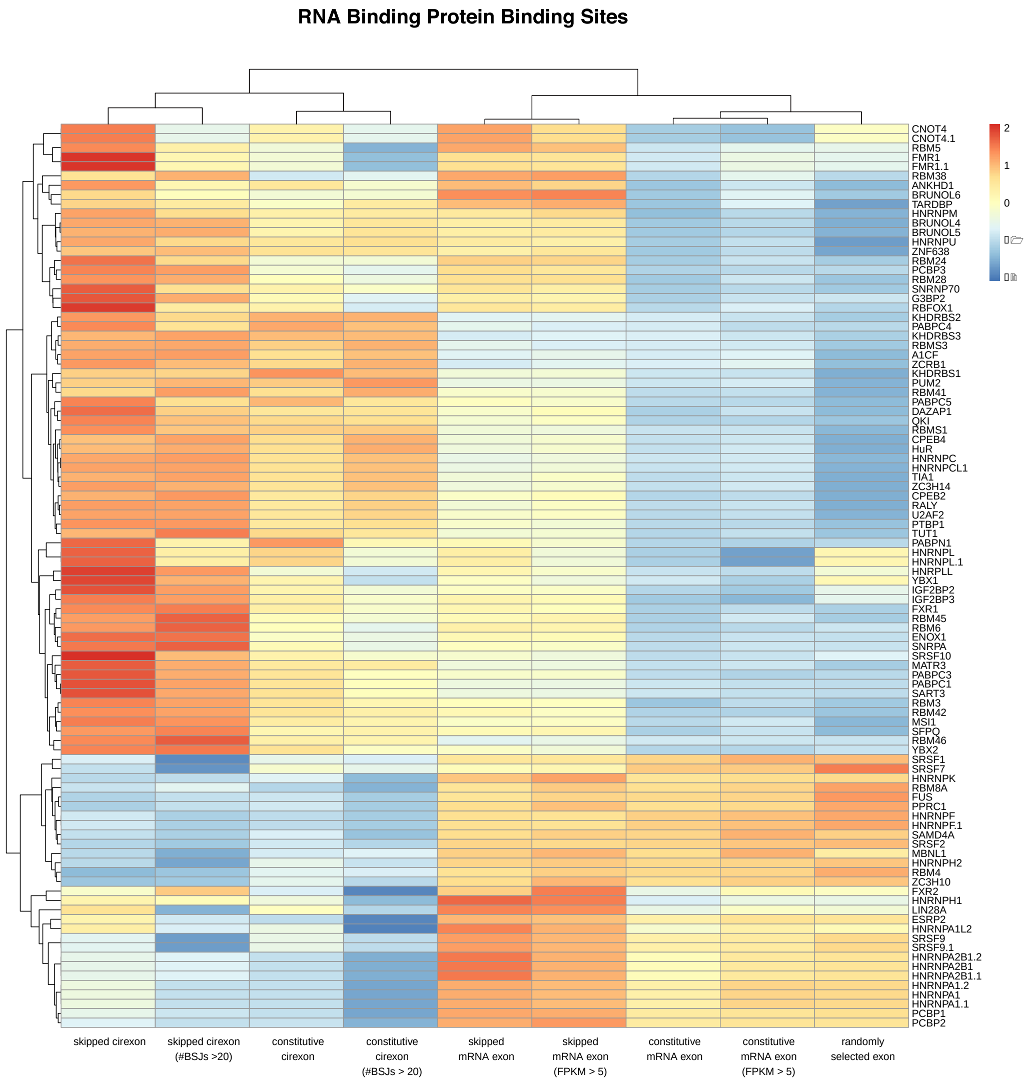

Supplementary Figure 18. Comparison of binding site density of RNA binding proteins on skipped cirexons, constitutive cirexons, skipped mRNA exons in HeLa cells and randomly selected annotated exons. The binding numbers were normalized by corresponding exon length to calculate average binding density, which was shown in the heatmap after Z-score normalization.

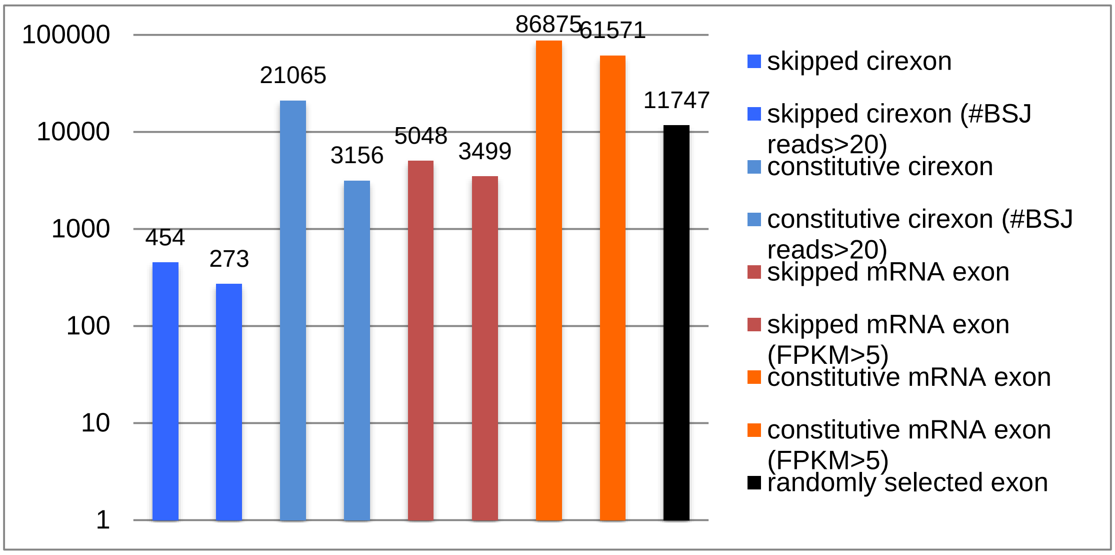

Supplementary Figure 19. Total number of exons in each group used for splicing factor binding site prediction. Colors of each group correspond to those in Fig 3A.

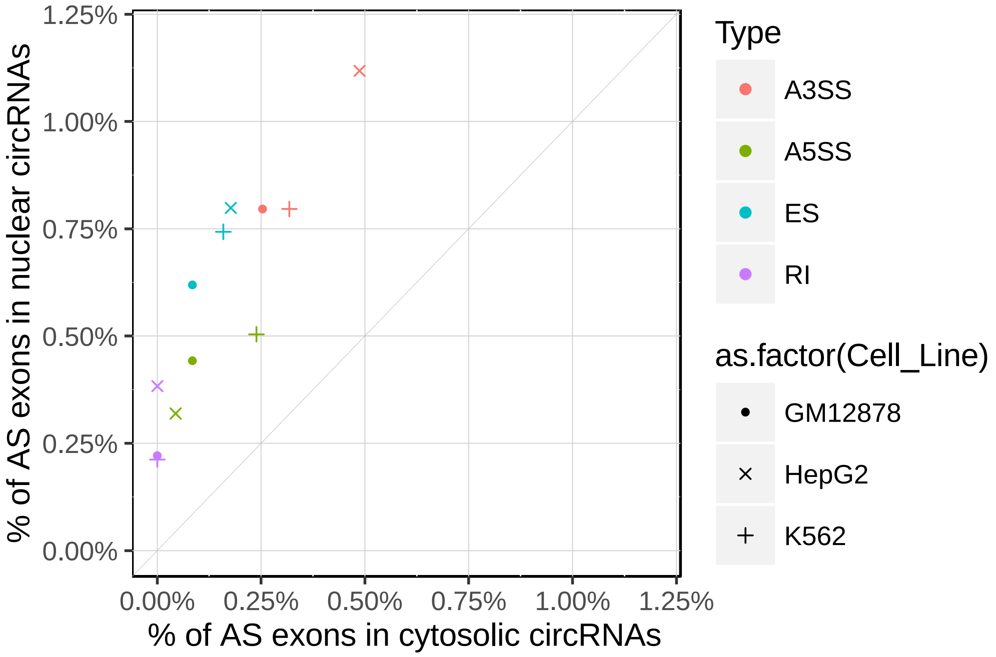

Supplementary Figure 20. A comparison between nucleus and cytoplasm for percentage of four types of alternatively spliced cirexons in HepG2, K562 and GM12878 cell lines. All of the three cell lines have comparable sequencing data size in nucleus and cytoplasm.

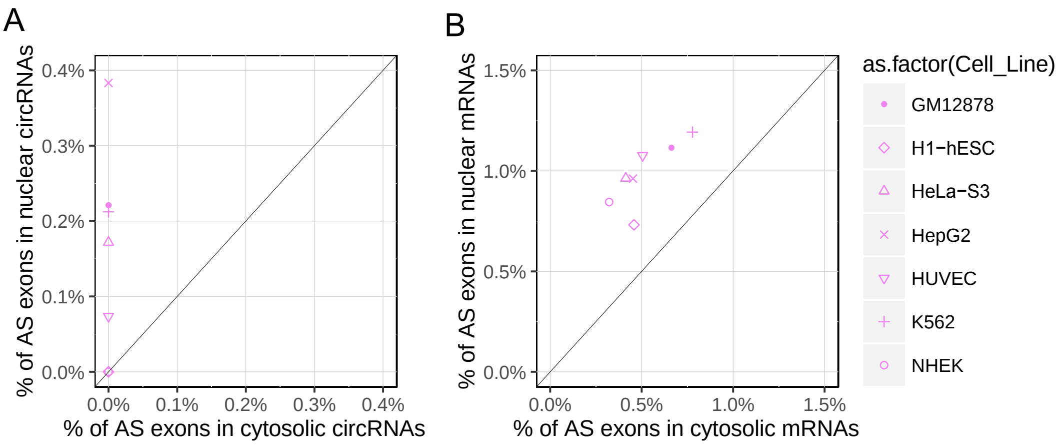

Supplementary Figure 21. A comparison between nucleus and cytoplasm for percentage of retained intron in seven cell lines. ((A) percentage of retained intron in circRNAs; (B) percentage of retained intron in mRNAs.

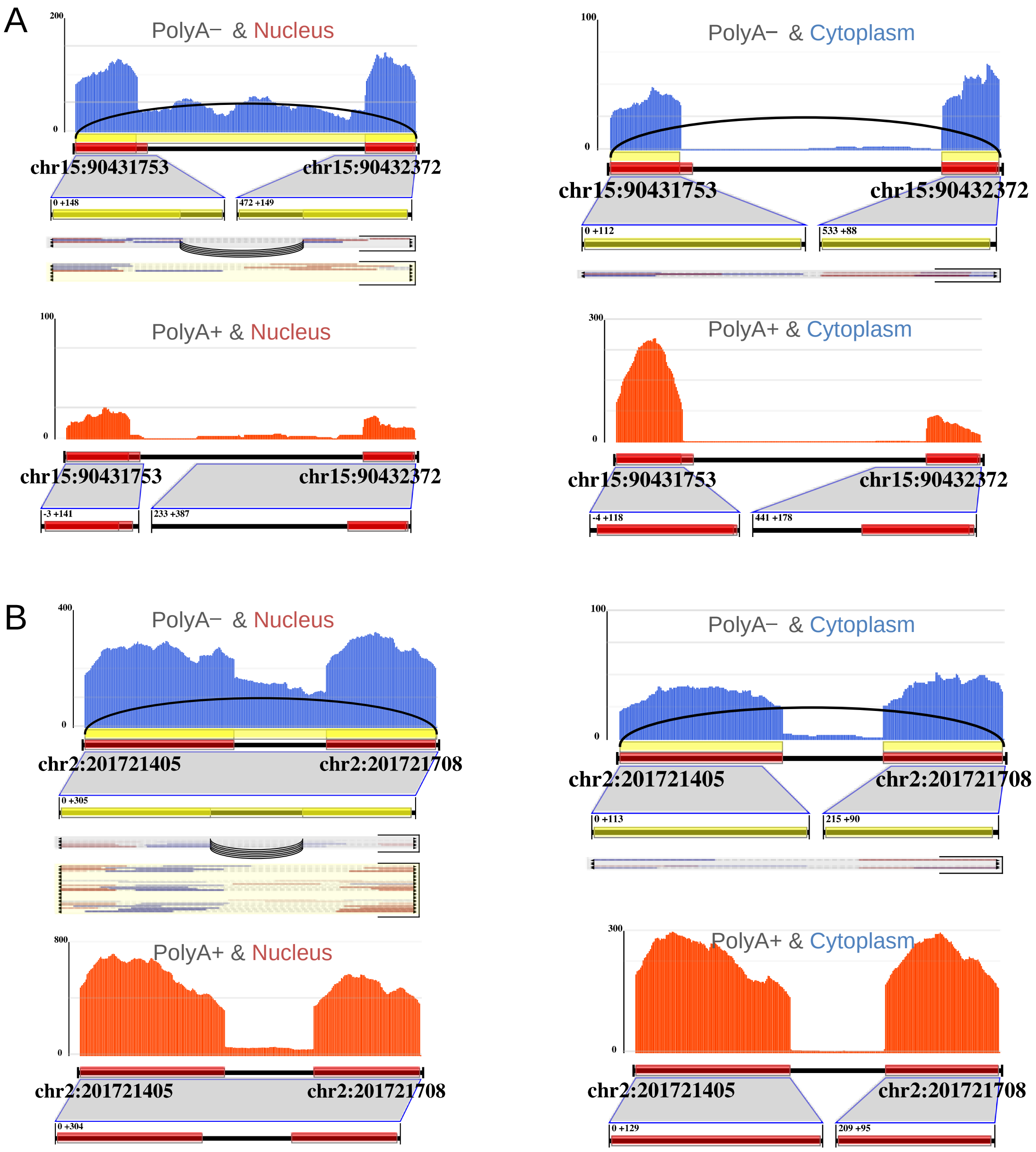

Supplementary Figure 22. Examples of intron retention within circRNA observed in poly(A)- sequencing data set of nucleus but not in cytoplasm. Sequencing depths of mRNA observed in corresponding poly(A) selected sequencing data sets of both nucleus and cytoplasm are shown. (A) Retained intron 2 in circAP3S2; (B) Retained intron 8 in circCLK1.

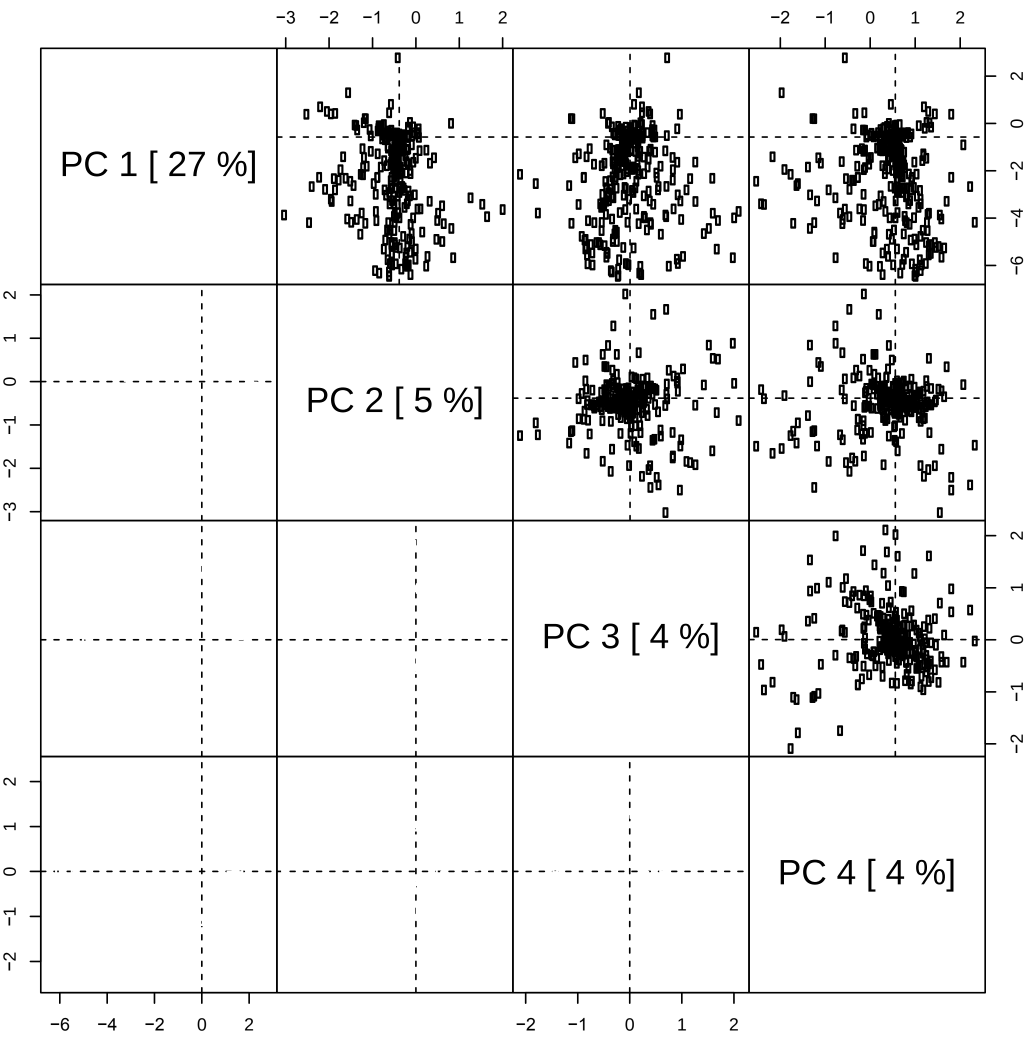

Supplementary Figure 23. Principal component analysis based on the Ψ values of the AS cirexons in fruit fly. Each dot represents a sample and its color corresponds to the sample type on the top of Figure 4A. The first four principal components were displayed.

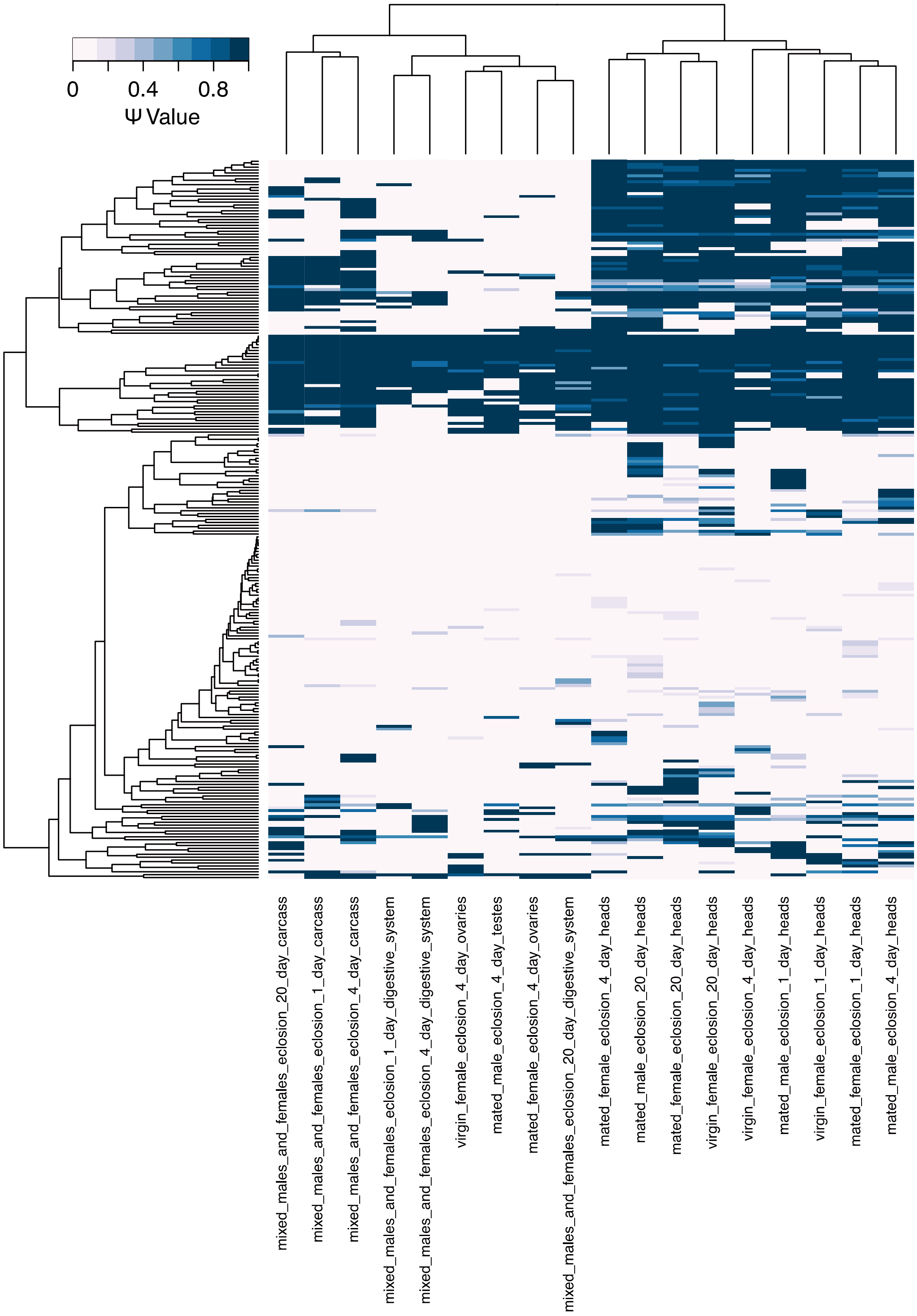

Supplementary Figure 24. Relative abundance of alternatively spliced cirexons in 18 fruit fly eclosion samples.

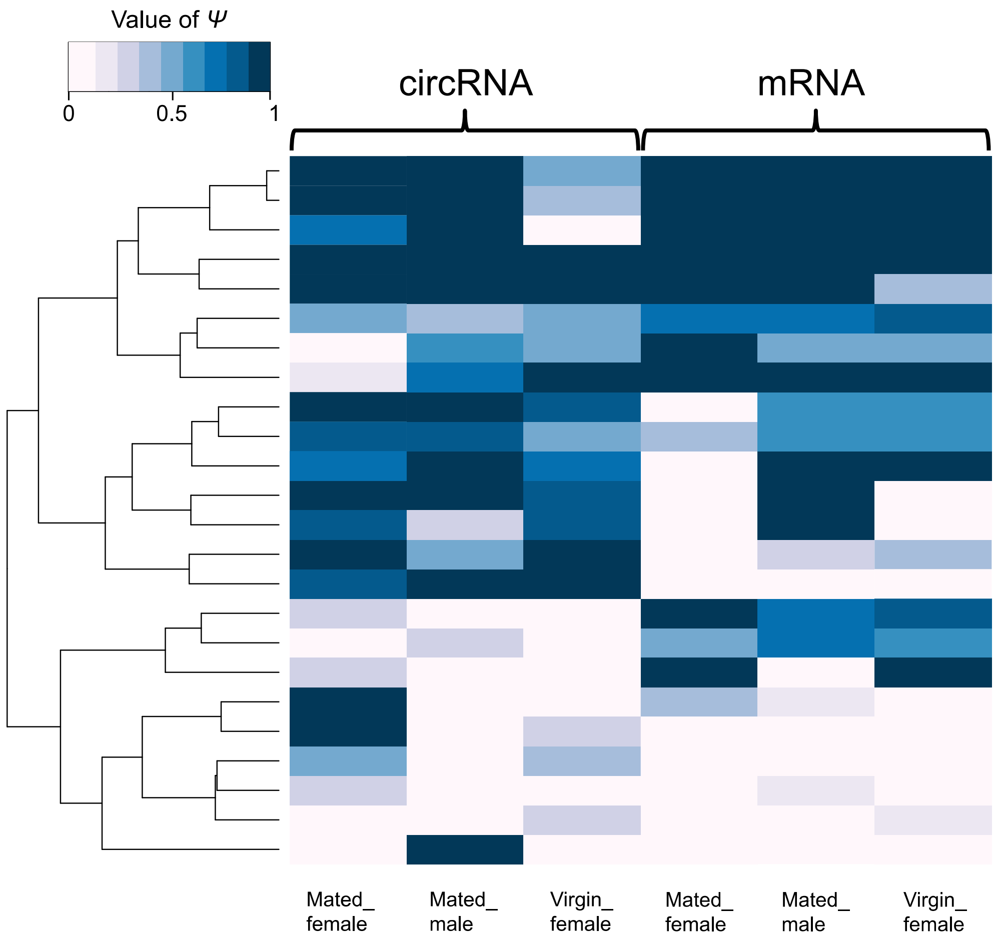

Supplementary Figure 25. Relative abundance of skipped cirexons detected in circRNAs compared with corresponding mRNAs in three fruit fly samples (eclosion + 1 day, heads).

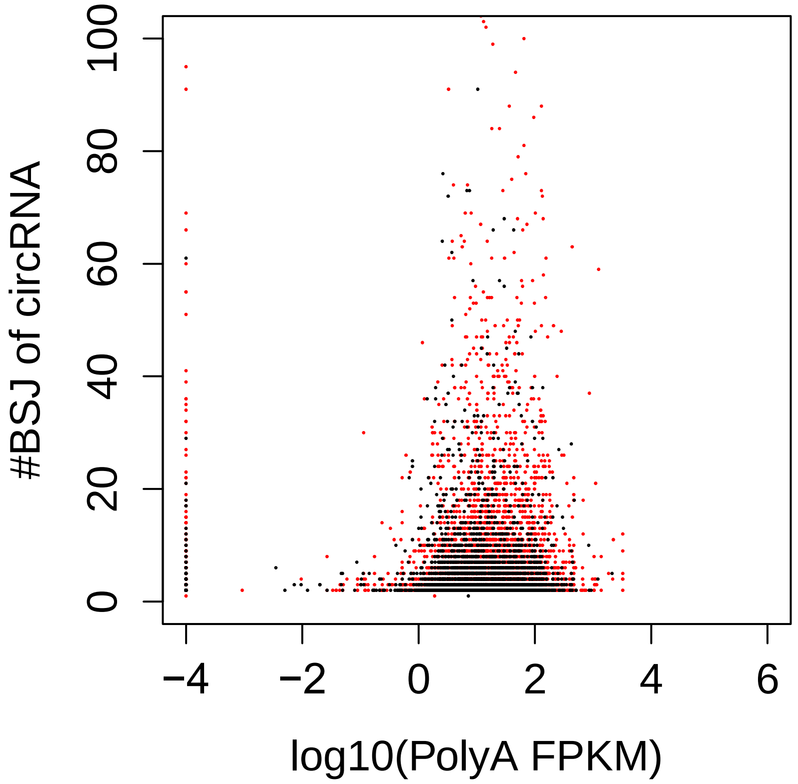

Supplementary Figure 26. Number of BSJ reads for circRNAs and FPKM of the corresponding linear RNAs in HEK293 and HeLa cell lines. Red dot: HeLa cell line; Black dot: HEK293 cell line.

Supplementary Table 1. Data sets of human samples used in this study for cirexon/exon detection

Cell type	Treatment	Read	Insert size (bp)	Data (Gb)	# BSJs	# cirexons	# ICFs	Data source (SRA accession #)
**HeLa**	RiboMinus	PE100	148.8±48.6	21.5	6,606	11,325	921	This study (SRR1637089, SRR1637090 & SRR3476958)
**HeLa**	RiboMinus & RNase R+	PE100	224.6±97.2	17.6	15,631	33,241	2,755	This study (SRR1636985, SRR1636986 & SRR3476956)
**HeLa**	PolyA+	PE100	216.9±109.2	2.9	n/a	n/a	n/a	This study (SRR3479116)
**HEK293**	RiboMinus	PE150	236±86.7	9.3	3,916	8,847	499	This study (SRR3479243)
**HEK293**	RiboMinus & RNase R+	PE150	250.3±103.2	12.7	13,465	33,650	2,453	This study (SRR3479244)
**HEK293**	PolyA+	PE150	268.7±103.5	3.9	n/a	n/a	n/a	This study (SRR3479143)
**Hs68**	RiboMinus	PE100	103.3±20.3	31.4	2,532	3,916	659	Jeck et al., 2013 (SRR444655)
**Hs68**	RiboMinus	PE100	174.6±65.7	41.3	4,387	7,475	1,089	Jeck et al., 2013 (SRR444975)
**Hs68**	RiboMinus & RNase R+	PE100	181.89±52.7	40.0	26,685	54,785	6,735	Jeck et al., 2013 (SRR445016)
**Hs68**	RiboMinus & RNase R+	PE100	179.2±66.3	31.7	26,537	53,974	5,702	Jeck et al., 2013 (SRR444974)

Supplementary Table 2. Exon skipping in top 50 most abundant circRNAs of HeLa cells.

circRNA	cirexon	Psi value	#BSJ reads
**chr9:138773479|138774924**	138774061:138774274	0.032	1275
**chr9:138773479|138774924**	138774061:138774222	0.04	1275
**chr2:61749746|61761038**	61752627:61752656	0.012	468
**chr2:61749746|61761038**	61752405:61752464	0.012	468
**chr2:61749746|61761038**	61756201:61756294	0.005	468
**chr2:61749746|61761038**	61752405:61752468	0.279	468
**chr2:61749746|61761038**	61752177:61752238	0.012	468
**chr21:46548317|46554747**	46552130:46552221	0.008	441
**chr21:46548317|46554747**	46553474:46553580	0.011	441
**chr3:196118684|196129890**	196127500:196127603	0.018	311
**chr3:196118684|196129890**	196120421:196120490	0.019	311
**chr3:196118684|196129890**	196127272:196127603	0.018	311
**chr3:196118684|196129890**	196120425:196120490	0.975	311
**chr7:99621042|99621930**	99621175:99621555	0.975	285
**chr15:25650608|25657118**	25652214:25652379	0.019	271
**chr15:25650608|25657118**	25653719:25653831	0.019	271
**chr15:25650608|25657118**	25652214:25652284	0.556	271
**chr15:25650608|25657118**	25653767:25653831	0.481	271
**chr15:25650608|25657118**	25652214:25652409	0.111	271
**chr21:17135210|17138460**	17136557:17136616	0.206	260
**chr21:17135210|17138460**	17137101:17137203	0.4	260
**chr21:17135210|17138460**	17137124:17137203	0.257	260
**chr21:17135210|17138460**	17137093:17137203	0.029	260
**chr3:149563798|149639014**	149619859:149619949	0.895	243
**chr5:32135678|32143986**	32139658:32139818	0.02	209
**chr12:109046048|109048186**	109046814:109046957	0.069	207
**chr12:109046048|109048186**	109046814:109046836	0.03	207
**chr8:128152989|128186609**	128176683:128176771	0.029	184
**chr8:128152989|128186609**	128174167:128174329	0.81	184
**chr8:128152989|128186609**	128153719:128153816	0.951	184
**chr8:128152989|128186609**	128161042:128161159	0.286	184
**chr8:128152989|128186609**	128161042:128161163	0.143	184
**chr8:128152989|128186609**	128181143:128181362	0.171	184
**chr2:61749746|61753656**	61752177:61752201	0.015	180
**chr2:61749746|61753656**	61752405:61752468	0.776	180
**chr2:61749746|61753656**	61752627:61752694	0.03	180
**chr2:61749746|61753656**	61752177:61752238	0.015	180
**chr2:61749746|61753656**	61752627:61752709	0.03	180
**chr8:128152989|128161917**	128161042:128161163	0.2	174
**chr8:128152989|128161917**	128161042:128161159	0.52	174
**chr8:128152989|128161917**	128153719:128153816	0.855	174
**chr8:141874411|141900868**	141889570:141889736	0.973	172
**chr4:186168448|186188309**	186176837:186176896	0.449	169
**chr3:142455221|142467302**	142462327:142462428	0.667	167
**chr9:96233423|96238620**	96236515:96236612	0.029	166
**chr12:116668338|116675510**	116674626:116674695	0.66	166
**chr12:116668338|116675510**	116669864:116669961	0.586	166
**chr10:7285520|7327916**	7318854:7318951	0.931	166
**chr21:38792601|38794168**	38793237:38793297	0.038	160
**chr3:31917925|31921322**	31920922:31921045	0.612	158
**chr3:125032152|125050082**	125033781:125033897	0.08	154

**chr21:16386665|16415895**	16387466:16387546	0.187	152
**chr1:117944808|117984947**	117983937:117984132	0.026	148
**chr5:138614016|138614818**	138614254:138614429	0.509	148
**chr22:46125305|46136418**	46132848:46132963	0.137	146
**chr20:57014001|57016139**	57014202:57014364	0.37	145
**chr9:114148657|114154104**	114152264:114152381	0.68	144
**chr17:80858527|80869665**	80861281:80861354	0.937	141
**chr17:80858527|80869665**	80865637:80865697	0.958	141
**chr5:179688684|179707608**	179706796:179707036	0.64	126
**chrX:24190832|24197887**	24191013:24191050	0.03	124
**chrX:24190832|24197887**	24193506:24193560	0.135	124
**chrX:24190832|24197887**	24191724:24191840	0.059	124
**chr5:176370336|176385155**	176378475:176378578	0.949	115
**chr7:24663285|24708279**	24690104:24690331	0.333	115
**chr7:24663285|24708279**	24689231:24689383	0.333	115
**chr19:3660964|3661999**	3661371:3661524	0.424	112
**chr11:128993341|129034322**	128993783:128993824	0.903	111
**chr11:128993341|129034322**	128997107:128997200	0.235	111
**chr20:30954187|30959677**	30957627:30957686	0.046	110
**chr13:61013822|61041513**	61018817:61018882	0.871	109
**chr13:61013822|61041513**	61034514:61034674	0.935	109
**chr13:61013822|61041513**	61018821:61018882	0.097	109
**chr10:93185038|93221941**	93221011:93221113	0.85	106
**chr9:33953283|33963789**	33956077:33956144	0.644	106
**chr6:159004986|159010814**	159007791:159007882	0.048	105
**chr2:240929491|240946787**	240942466:240942557	0.086	101
**chr2:240929491|240946787**	240942466:240942553	0.029	101
**chr2:240929491|240946787**	240939600:240939804	0.296	101
**chr2:240929491|240946787**	240945165:240945258	0.024	101
**chr9:98740343|98766983**	98759528:98759695	0.078	101
**chr21:42598193|42629253**	42599114:42599149	0.02	97
**chr21:42598193|42629253**	42622679:42622828	0.882	97
**chr12:19615444|19626289**	19619682:19619784	0.355	97
**chr14:97299804|97327072**	97322467:97322587	0.5	96
**chr9:126519982|126641300**	126554866:126554909	0.872	95
**chr11:46098305|46113774**	46112006:46112117	0.3	93
**chr21:46538479|46554747**	46540709:46540874	0.889	93
**chrX:53672263|53681075**	53675516:53675593	0.119	93
**chr8:141856359|141900868**	141889570:141889736	0.455	91
**chr8:141856359|141900868**	141874411:141874498	0.889	91

Supplementary Table 3. **Alternative 5'/3' splicing site events in top 50 most abundant circRNAs of HeLa cells.**

circRNA	cirexon	Psi value	#BSJ reads
**chr9:138773479|138774924**	138774061:138774274	0.032	1275
**chr9:138773479|138774924**	138774061:138774222	0.04	1275
**chr9:138773479|138774924**	138774854:138774924	0.008	1275
**chr9:138773479|138774924**	138774662:138774924	0.992	1275
**chr1:247318868|247323115**	247318868:247318997	0.958	1055
**chr1:247318868|247323115**	247318868:247318927	0.042	1055
**chr10:7318854|7327916**	7318854:7318951	0.995	656
**chr10:7318854|7327916**	7325866:7325921	0.99	656
**chr10:7318854|7327916**	7318854:7318934	0.005	656
**chr10:7318854|7327916**	7325866:7326112	0.01	656
**chr2:61749746|61761038**	61752405:61752468	0.279	468
**chr2:61749746|61761038**	61753555:61753656	0.988	468
**chr2:61749746|61761038**	61752177:61752238	0.012	468
**chr2:61749746|61761038**	61753597:61753656	0.012	468
**chr2:61749746|61761038**	61752405:61752464	0.012	468
**chr5:619105|620376**	619105:619185	0.007	405
**chr5:619105|620376**	620184:620376	0.932	405
**chr5:619105|620376**	619105:619232	0.993	405
**chr5:619105|620376**	620251:620376	0.068	405
**chr3:196118684|196129890**	196127500:196127603	0.018	311
**chr3:196118684|196129890**	196127272:196127603	0.018	311
**chr3:196118684|196129890**	196120421:196120490	0.019	311
**chr3:196118684|196129890**	196120425:196120490	0.975	311
**chr15:25650608|25657118**	25653767:25653831	0.481	271
**chr15:25650608|25657118**	25652214:25652409	0.111	271
**chr15:25650608|25657118**	25652214:25652379	0.019	271
**chr15:25650608|25657118**	25652214:25652284	0.556	271
**chr15:25650608|25657118**	25653719:25653831	0.019	271
**chr5:167915607|167921655**	167919664:167919826	0.056	264
**chr5:167915607|167921655**	167920924:167921007	0.056	264
**chr5:167915607|167921655**	167919664:167919852	0.889	264
**chr5:167915607|167921655**	167919664:167919796	0.056	264
**chr5:167915607|167921655**	167920899:167921007	0.944	264
**chr21:17135210|17138460**	17137124:17137203	0.257	260
**chr21:17135210|17138460**	17138344:17138460	0.029	260
**chr21:17135210|17138460**	17137101:17137203	0.4	260
**chr21:17135210|17138460**	17138316:17138460	0.829	260
**chr21:17135210|17138460**	17137093:17137203	0.029	260
**chr21:17135210|17138460**	17138321:17138460	0.143	260
**chr3:149563798|149639014**	149613260:149613357	0.4	243
**chr3:149563798|149639014**	149613260:149613347	0.6	243
**chr5:32135678|32143986**	32143907:32143986	0.014	209
**chr5:32135678|32143986**	32143855:32143986	0.986	209
**chr12:109046048|109048186**	109046814:109046957	0.069	207
**chr12:109046048|109048186**	109048082:109048186	0.99	207
**chr12:109046048|109048186**	109046814:109046836	0.03	207
**chr12:109046048|109048186**	109048047:109048186	0.01	207
**chr8:128152989|128186609**	128156008:128156164	0.2	184
**chr8:128152989|128186609**	128156008:128156104	0.8	184
**chr8:128152989|128186609**	128161042:128161159	0.286	184
**chr8:128152989|128186609**	128161042:128161163	0.143	184
**chr2:61749746|61753656**	61752627:61752694	0.03	180
**chr2:61749746|61753656**	61752627:61752709	0.03	180
**chr2:61749746|61753656**	61752177:61752238	0.015	180
**chr2:61749746|61753656**	61752177:61752201	0.015	180
**chr8:128152989|128161917**	128161042:128161163	0.2	174

**chr8:128152989|128161917**	128161042:128161159	0.52	174
**chr8:128152989|128161917**	128156008:128156164	0.182	174
**chr8:128152989|128161917**	128156008:128156104	0.636	174
**chr8:128152989|128161917**	128156008:128156139	0.182	174
**chr12:69644909|69656342**	69653824:69653977	0.947	172
**chr12:69644909|69656342**	69653824:69653852	0.053	172
**chr18:19345733|19359646**	19358064:19358130	0.848	170
**chr18:19345733|19359646**	19358007:19358130	0.152	170
**chr10:7285520|7327916**	7325866:7326112	0.04	166
**chr10:7285520|7327916**	7325866:7325921	0.96	166
**chr9:140705913|140712590**	140707481:140707625	0.053	155
**chr9:140705913|140712590**	140707458:140707625	0.947	155
**chr3:125032152|125050082**	125042232:125042288	0.12	154
**chr3:125032152|125050082**	125042153:125042288	0.88	154
**chr9:33971649|33973235**	33971649:33971752	0.956	152
**chr9:33971649|33973235**	33971649:33971735	0.044	152
**chr12:46622936|46637097**	46622936:46623044	0.037	151
**chr12:46622936|46637097**	46622936:46623051	0.963	151
**chr2:58449077|58459247**	58453863:58453910	0.025	148
**chr2:58449077|58459247**	58453863:58453919	0.975	148
**chr22:46125305|46136418**	46134641:46134719	0.043	146
**chr22:46125305|46136418**	46134611:46134719	0.957	146
**chr22:46125305|46136418**	46125305:46125445	0.036	146
**chr22:46125305|46136418**	46125305:46125470	0.964	146
**chr22:46125305|46136418**	46136249:46136418	0.929	146
**chr22:46125305|46136418**	46136273:46136418	0.071	146
**chr9:74838036|74846097**	74842863:74842956	0.071	145
**chr9:74838036|74846097**	74842859:74842956	0.929	145
**chr9:114148657|114154104**	114152264:114152381	0.68	144
**chr2:72958136|72960247**	72958136:72958175	0.013	140
**chr2:72958136|72960247**	72958136:72958181	0.987	140
**chr2:72958136|72960247**	72960112:72960247	0.024	140
**chr2:72958136|72960247**	72960200:72960247	0.976	140
**chr2:58311224|58316858**	58312016:58312085	0.565	135
**chr2:58311224|58316858**	58312016:58312134	0.435	135
**chr7:24663285|24708279**	24681335:24681457	0.5	115
**chr7:24663285|24708279**	24681335:24681487	0.5	115
**chr15:41988273|41991357**	41988273:41988388	0.056	113
**chr15:41988273|41991357**	41988273:41989221	0.944	113
**chr19:3660964|3661999**	3660964:3661106	0.029	112
**chr19:3660964|3661999**	3660964:3661044	0.029	112
**chr19:3660964|3661999**	3660964:3661081	0.941	112
**chr7:148543562|148544397**	148543562:148543690	0.958	111
**chr7:148543562|148544397**	148543562:148543679	0.042	111
**chr11:128993341|129034322**	128997107:128997200	0.235	111
**chr13:61013822|61041513**	61018821:61018882	0.097	109
**chr13:61013822|61041513**	61018817:61018882	0.871	109
**chr6:159004986|159010814**	159010680:159010814	0.048	105
**chr6:159004986|159010814**	159010704:159010814	0.952	105
**chr1:9991949|9994918**	9994830:9994918	0.06	102
**chr1:9991949|9994918**	9994820:9994918	0.94	102
**chr14:102506573|102507010**	102506573:102506747	0.615	101
**chr14:102506573|102507010**	102506573:102506776	0.385	101
**chr2:240929491|240946787**	240942466:240942557	0.086	101
**chr2:240929491|240946787**	240942466:240942553	0.029	101
**chr14:97299804|97327072**	97326894:97327072	0.7	96
**chr14:97299804|97327072**	97326984:97327072	0.3	96
**chr9:126519982|126641300**	126531793:126531842	0.944	95
**chr9:126519982|126641300**	126531793:126531838	0.056	95
**chr21:46538479|46554747**	46540709:46540874	0.889	93
**chr8:141856359|141900868**	141900642:141900868	0.875	91

**chr8:141856359|141900868**	141900764:141900868	0.125	91
**chr16:69404386|69406258**	69404386:69404647	0.13	85
**chr16:69404386|69406258**	69404386:69404532	0.87	85
**chr8:18656805|18662408**	18662032:18662132	0.397	85
**chr8:18656805|18662408**	18662032:18662112	0.603	85
**chr1:59180504|59184420**	59181166:59181313	0.917	83
**chr1:59180504|59184420**	59181199:59181313	0.083	83
**chr1:59180504|59184420**	59183429:59183592	0.75	83
**chr1:59180504|59184420**	59183429:59183596	0.125	83
**chr10:74468041|74475660**	74468041:74468128	0.111	82
**chr10:74468041|74475660**	74475520:74475660	0.067	82
**chr10:74468041|74475660**	74475612:74475660	0.933	82
**chr10:74468041|74475660**	74468041:74468114	0.889	82
**chr17:1746097|1756483**	1747214:1747321	0.062	80
**chr17:1746097|1756483**	1747214:1747292	0.938	80
**chr6:159001972|159010814**	159010680:159010814	0.056	80
**chr6:159001972|159010814**	159010704:159010814	0.944	80
**chr1:21377359|21415706**	21386239:21386287	0.061	79
**chr1:21377359|21415706**	21386218:21386287	0.156	79
**chr4:77055328|77065626**	77057339:77057559	0.5	78
**chr4:77055328|77065626**	77057339:77057565	0.5	78

Supplementary Table 4. Intron retention in circRNAs of HeLa cells.

circRNA	cirexon	Psi value	#BSJ reads
**chr9:138773479|138774924**	138773479:138774924	0.168	1275
**chr5:619105|620376**	619105:620376	0.035	405
**chr7:99621042|99621930**	99621042:99621555	0.931	285
**chr12:120592774|120593523**	120592774:120593234	0.624	231
**chr8:37623044|37623873**	37623044:37623264	0.288	150
**chr15:59204762|59209198**	59204762:59205895	0.023	122
**chr19:3660964|3661999**	3660964:3661999	0.086	112
**chr10:105197772|105198565**	105197772:105198565	0.07	64
**chr10:74474869|74475660**	74474869:74475660	0.231	58
**chr11:128993341|128994789**	128993783:128994789	0.077	46
**chr1:47761437|47767420**	47765577:47765824	0.482	31
**chr19:4941540|4941943**	4941540:4941943	0.559	20
**chr2:201721405|201721708**	201721405:201721708	0.531	17
**chr1:1192372|1192690**	1192372:1192690	0.383	15
**chr19:8987045|8987334**	8987045:8987334	0.466	11
**chr2:39564061|39564722**	39564061:39564722	0.467	9

Supplementary Table 5. Increased miRNA binding sites within skipped cirexons in Hs68 cell line.

miRNA	cirexon(start:end) within circRNA(chr:start|end)	Length_ miRNA	Length_ cirexon	Postions_binding_ sites
**hsa-miR-7851-3p**	245185471:245185598 chr1:245165423|245222760	22	128	86 54 1
**hsa-miR-7114-5p**	122279976:122280258 chr10:122273423|122280607	21	283	173 72 213
**hsa-miR-6765-5p**	120277289:120277546 chr11:120276827|120278532	25	258	205 77 48
**hsa-miR-3186-3p**	114173850:114174116 chr13:114164553|114175048	21	267	94 167 240
**hsa-miR-3614-3p**	114173850:114174116 chr13:114164553|114175048	23	267	83 156 227
**hsa-miR-4462**	114173850:114174116 chr13:114164553|114175048	23	267	237 91 164
**hsa-miR-4802-3p**	114173850:114174116 chr13:114164553|114175048	23	267	91 164 237
**hsa-miR-6514-5p**	114173850:114174116 chr13:114164553|114175048	23	267	89 162 235
**hsa-miR-4530**	27250927:27251320 chr17:27248706|27254081	18	394	218 304 272
**hsa-miR-15a-5p**	42232117:42232306 chr17:42231924|42232779	22	190	158 44 118
**hsa-miR-15b-5p**	42232117:42232306 chr17:42231924|42232779	22	190	158 120 44
**hsa-miR-497-5p**	42232117:42232306 chr17:42231924|42232779	21	190	119 163 45
**hsa-miR-4725-3p**	234298495:234298687 chr2:234296903|234299129	22	193	89 58 160
**hsa-miR-4725-3p**	234298541:234298687 chr2:234296903|234299129	22	147	43 12 114
**hsa-miR-6860**	372617:372746 chr5:353845|376831	22	130	49 8 78
**hsa-miR-330-5p**	136791245:136791449 chr9:136790965|136804341	22	205	47 159 178
**hsa-miR-326**	136791245:136791449 chr9:136790965|136804341	20	205	46 164 180

Supplementary Table 6. circRNA producing loci with AS events validated in RT-PCR of this study and corresponding outward-facing primers.

chr	start position	end position	outward-facing primer		isoform length (bp)	PCR & Sanger
			P1	P2		
chr1	117944808	117984947	GTGCTACTTTTCCTAATAGAAC	TCTCTTATATGCATCAAGTCTA	844	y
chr1	117944808	117984947	TTGGGCAATGGTGAATTTGAA	AGGTGTGTTAAAGGCAGGAA	648	y
chr1	231090079	231097049	GTTCGCAGTCTGAGTCATGGAA	ACCCTGTGACTCCTTGCTTT	382	y
chr1	231090079	231097049	GTTCGCAGTCTGAGTCATGGAA	ACCCTGTGACTCCTTGCTTT	304	y
chr1	231090079	231097049	GTTCGCAGTCTGAGTCATGGAA	ACCCTGTGACTCCTTGCTTT	242	y
chr1	1192372	1192690	GAGCTTCATGGTGGAGAAGGG	CGCAGCCAGAAAGCCTCC	319	n
chr1	1192372	1192690	GCAGTATAGAGACGTCGGACT	TGTCCGGGTGGAAATCCGTGA	242	y
chr10	74474869	74475660	CAGTGACATCTTAGCTGCTA	CGGTATCTTGCCAATCACTGTA	165	y
chr10	74474869	74475660	CAGGGGAAACTTGAACATTCG	CACCAATACCTAAGACAGAGCAG	257	y
chr11	86267595	86270865	AGTGACCTGGACAAGAGT	TGCTGCCGCTCGTAATATCT	344	y
chr11	86267595	86270865	GACGTGGAGAAGTTCATGCCAA	TCGTTCCGGTCTTGGAGTGTCA	326	y
chr11	86267595	86270865	GTGACCTGGACAAGTACATCATT	TGCTGCCGCTCGTAATATCT	284	y
chr12	19615444	19626289	CGTTCAACTCCAGCAATGATGAAT	ACTGGAAATTGTGCTGTCTACAT	419	y
chr12	19615444	19626289	CGTTCAACTCCAGCAATGATGAAT	ACTGGAAATTGTGCTGTCTACAT	316	y
chr12	28378728	28412375	CCAAGTTCCAGGATTAATTCTG	GTCACATAAAGGAGGGTTAGAA	409	y
chr12	28378728	28412375	CCAAGTTCCAGGATTAATTCTG	GTCACATAAAGGAGGGTTAGAA	332	y
chr12	28378728	28412375	CCAAGTTCCAGGATTAATTCTG	GTCACATAAAGGAGGGTTAGAA	272	n
chr12	109046048	109048186	AGTGGACGTACGGGGATTCAT	ACACATTCAGCAGCAAGGAG	251	y
chr12	109046048	109048186	TGAACAATGACGGCAATCTTGT	GAGGGGACTTGATGTTAACAAA	395	n
chr12	109046048	109048186	AGTGGACGTACGGGGATTCAT	ACACATTCAGCAGCAAGGAG	274	y
chr12	116668338	116675510	TTGCAGACAGCGGATGAAACTTA	ACTGCAGGTGGTATTAAATTTGTG	328	y
chr12	116668338	116675510	AAGCTAAAAATCAGGCAACACC	GCATCTGATATCTGGAAACTGAA	398	y
chr12	116668338	116675510	AAGCTAAAAATCAGGCAACACC	GCATCTGATATCTGGAAACTGAA	496	y
chr12	116668338	116675510	TTGCAGACAGCGGATGAAACTTA	ACATCATGAACTGCAGGAGTG	426	y
chr12	116668238	116675510	CTGCTGCAAAATGGTATCATTC	ATTGGTATCACAGTGCACCT	338	y
chr12	116668238	116675510	CTGCTGCAAAATGGTATCATTC	ATTGGTATCACAGTGCACCT	408	y
chr12	116668238	116675510	CTGCTGCAAAATGGTATCATTC	ATTGGTATCACAGTGCACCT	436	y
chr13	33091994	33101669	TCTGTATCCTTTTCCTATGGC	TGGTGGAAATTTGATCCTGA	438	y
chr13	33091994	33101669	TCTGTATCCTTTTCCTATGGC	TGGTGGAAATTTGATCCTGA	388	y
chr14	102506573	102507010	GTGCTGGAATTCTGCATCGTAGGT	TGAGATTGTCCTGAGTGCTGGCT	280	n
chr14	102506573	102507010	GTGCTGGAATTCTGCATCGTAGGT	TGAGATTGTCCTGAGTGCTGGCT	251	n
chr19	3660964	3661999	CAGGTGCATAGGTCTTGAAGCG	TTCCGGGAGCTCTTTGGGAT	249	y
chr19	3660964	3661999	CAGGTGCATAGGTCTTGAAGCG	TTCCGGGAGCTCTTTGGGAT	403	y
chr19	13039156	13039661	GCTCTCAATGAAGTTATCAGTCGGC	TCTGGAACTTTGACGCCCTCTT	300	y
chr19	13039156	13039661	GCTCTCAATGAAGTTATCAGTCGGC	TCTGGAACTTTGACGCCCTCTT	338	y
chr2	58311224	58316858	TAACGGGCCATTTTCTTGA	GCTACTTCCATATATGTGCTT	456	y
chr2	58311224	58316858	GACCAGATCCATAAAACAGA	GACTGAATTCAAGGGAAGAA	407	y
chr21	16386665	16415895	TGTCATCCGGAGTCTTCAGATT	AGAGCTACTTTTCAACAGCCTT	203	y
chr21	16386665	16415895	ACATTCCAGAAAACGCAAATTC	GTCTGGCTTCTTGCACTCAG	284	y
chr22	17525763	17528316	GCAACTGTGGATGAAGAGGGCT	TTCGGCTGTGTTCTCTGGGTTA	231	y
chr22	17525763	17528316	GGTGAAGGCATTGATTGTCCTGA	AACGAGGGCAGAGGCTCATGAT	269	y
chr3	31917925	31921322	CGAGGCTTCTGGTGTTTGCTTT	TGCTGGTGGTGTACTCTGCTAAT	380	y
chr3	31917925	31921322	CGAGGCTTCTGGTGTTTGCTTT	TGCTGGTGGTGTACTCTGCTAAT	256	y

**chr3**	125032152	125050082	TGGTGAGGCATACTTCGATCTT	TTGCTGCAGATGAAGTGTTACA	683	y
**chr3**	125032152	125050082	TGGTGAGGCATACTTCGATCTT	TTGCTGCAGATGAAGTGTTACA	566	y
**chr3**	125032152	125050082	TGGTGAGGCATACTTCGATCTT	TTGCTGCAGATGAAGTGTTACA	487	y
**chr3**	142455221	142467302	ACGATGAGCAGCTAAAATGACA	CCCATGCAGTTGGCTGTGAAT	460	n
**chr3**	142455221	142467302	ACGATGAGCAGCTAAAATGACA	CCCATGCAGTTGGCTGTGAAT	358	n
**chr7**	99621042	99621930	GCATCTCAGGTCCATGAACTTG	AGTCCTCGAGCTTTGACCTTCAT	578	n
**chr7**	99621042	99621930	GCATCTCAGGTCCATGAACTTG	AGTCCTCGAGCTTTGACCTTCAT	197	n
**chr8**	37623044	37623873	CAGGTTTGGTTTTGCTGACCGC	GGTGATCGAGGCCTATGGACAT	220	y
**chr8**	37623044	37623873	TGGTTTTGCTGACCGCCACTAG	CCTGAAGCCCTTTGGAAGCAT	297	y
**chr8**	37623044	37623873	CAGGTTTGGTTTTGCTGACCGC	GGTGATCGAGGCCTATGGACAT	220	y
**chr8**	37623044	37623873	TGGTTTTGCTGACCGCCACTAG	CCTGAAGCCCTTTGGAAGCAT	297	y
**chr9**	33953283	33963789	GAGTTGGCTTCTGAGGCTTGACT	ATTCGCAACACAACAATCAGATGG	377	y
**chr9**	33953283	33963789	GAGTTGGCTTCTGAGGCTTGACT	ATTCGCAACACAACAATCAGATGG	309	y
**chr9**	134312007	134314448	AGCCAGTAGGACACGAAGGT	TCAGCTGTGCCCATGACTTTG	226	y
**chr9**	134312007	134314448	AGCCAGTAGGACACGAAGGT	TCAGCTGTGCCCATGACTTTG	176	y
**chr9**	33971649	33973235	TCTCCCTCTGCCACGTCCAAAT	AAGGCATGGGGACATTTAATCCT	159	y
**chr9**	33971649	33973235	TCTCCCTCTGCCACGTCCAAAT	AAGGCATGGGACTATTCAGATTCT	142	y
**chr9**	138773479	138774924	ATGAGCGTTCTGGGTCAGG	TGGTGTCCCTGTAAGGCGG	587	y
**chr9**	138773479	138774924	ACCCATAAAAATGTATCCCACCC	TGGTCCCCTTTCAGGATGAGG	639	y

Supplementary Table 7. circRNAs AS isoforms validated and quantified in qRT-PCR of this study and the corresponding primers used.

chr	start position	end position	outward-facing primer		isoform length (bp)
			P1	P2	
**chr19**	3660964	3661999	GCCAGATGATTACTTGACCACCTCCT	TGAGGTTGCTGCCTTCGCTGGGGAA	249
**chr19**	3660964	3661999	AGGAGGTGGTCAAGTAATCATCTGGC	TTCTTCCCCAGCTCTGTGACCAGT	403
**chr3**	31917925	31921322	GCAGCTCTCAGTTTAAACATCT	GGAAATGAATTCTAAGGTACTTCGT	256
**chr3**	31917925	31921322	CTCCAAACACAGCAAACTCTCA	GGAAATGAATTCTAAGGTACTTCGT	380
**chr9**	33971649	33973235	AAGGCATGGGACTATTCAGATTCT	CGTCCAAATCCAGTTCCCTCAT	142
**chr9**	33971649	33973235	AAGGCATGGGGACATTTAATCCTG	CGTCCAAATCCAGTTCCCTCAT	158
**chr1**	231090079	231097049	AGTAAATATCTAACTCTTCATTTGTGC	TTTTCAACAATGCTTCAGAGTCATC	382
**chr1**	231090079	231097049	TTCAAAAAGGATGACTCTGAAGCATT	CAGCACAAATGAAGAGTTAGGCTTA	304
**chr1**	231090079	231097049	CAAGACATAAGCAATAGCTAACTCTTC	TTTCAACAATGCTTCAGAGTCATCC	242

Supplementary Table 8. Significance of Mann–Whitney U tests after FDR-correction for comparison of splicing factor binding density between groups of exons.

	cirES~mES	cirES20~mES5	cirES~cirConst	cirES20~cirConst20	mES~mConst	mES5~mConst5	cirES~rand	cirES20~rand	mES~rand	mES5~rand
**FOX1**	4.3E-01	4.3E-01	8.5E-02	1.6E-01	7.1E-64	4.1E-35	2.0E-04	1.6E-02	6.6E-45	5.1E-31
**Tra2alpha**	2.4E-01	1.5E-01	8.1E-01	5.6E-01	5.2E-08	1.4E-04	7.8E-01	5.4E-01	1.5E-03	2.0E-03
**SRp55**	1.5E-01	4.5E-01	1.4E-02	9.4E-03	1.6E-17	3.0E-07	2.6E-04	1.7E-02	1.6E-13	6.4E-10
**Tra2beta**	5.9E-04	4.4E-03	7.1E-03	5.0E-01	6.7E-50	9.1E-32	1.8E-18	4.1E-12	9.0E-48	5.9E-36
**9G8**	4.5E-04	8.8E-04	7.8E-01	7.7E-01	4.8E-04	1.2E-01	2.9E-05	3.3E-05	3.4E-01	5.5E-02
**SC35**	3.4E-13	2.0E-10	7.5E-01	7.3E-01	2.4E-08	3.7E-04	4.2E-13	2.1E-10	3.4E-01	3.5E-01
**QK1**	8.8E-04	4.4E-03	2.0E-03	1.2E-02	8.4E-54	2.9E-38	3.5E-16	2.7E-11	4.9E-38	2.1E-29
**MBNL**	4.4E-02	1.1E-02	2.3E-02	5.6E-02	1.2E-21	2.7E-12	8.2E-01	4.2E-01	6.2E-09	8.6E-09
**NOVA1**	5.0E-01	7.6E-01	2.0E-03	1.6E-03	1.9E-37	1.4E-15	1.9E-01	2.4E-01	5.2E-11	7.3E-04
**hnRNPM**	1.4E-01	5.0E-01	7.1E-04	2.2E-02	8.4E-96	2.2E-53	4.4E-15	2.6E-08	1.9E-75	6.0E-59
**SF2ASF**	3.1E-06	2.0E-05	8.1E-01	7.3E-01	1.2E-07	1.7E-04	2.9E-05	2.3E-04	3.0E-02	6.6E-03
**SRp40**	6.5E-02	4.5E-02	2.0E-03	5.2E-03	1.4E-45	7.2E-21	5.4E-01	8.2E-01	3.2E-20	8.2E-12
**YB1**	2.7E-01	7.6E-01	1.2E-02	4.2E-02	1.9E-16	6.8E-10	4.2E-02	5.4E-01	2.6E-03	1.1E-01
**hnRNPA1**	6.8E-02	2.7E-01	1.5E-02	7.1E-03	7.3E-16	9.4E-09	7.7E-01	9.7E-01	4.1E-06	2.6E-05
**hnRNPU**	2.0E-10	1.4E-06	2.7E-06	4.2E-02	1.1E-78	1.9E-55	1.8E-42	1.7E-27	3.0E-88	7.6E-73
**hnRNPA2B1**	1.2E-02	1.8E-02	8.5E-02	3.5E-01	5.4E-30	4.7E-14	8.7E-01	5.7E-01	1.4E-13	7.3E-10
**PTB**	4.4E-03	7.6E-03	2.6E-03	1.4E-02	1.0E-97	4.7E-51	1.6E-01	7.7E-01	3.7E-48	3.5E-31
**hnRNPH1**	3.4E-04	2.0E-03	7.7E-01	5.8E-01	1.4E-01	8.5E-01	2.3E-06	9.6E-05	2.7E-02	5.4E-02
**hnRNPF**	1.1E-05	8.8E-04	5.5E-01	5.6E-01	4.3E-30	1.2E-14	2.6E-02	1.3E-01	2.3E-13	1.2E-10
**SRp20**	1.2E-04	5.9E-04	2.1E-02	1.5E-02	7.5E-26	3.1E-13	4.9E-03	2.0E-03	4.3E-05	3.7E-02

Supplementary Table 9. Intron retention events detected in ENCODE nucleus transcriptomic data and their Ψ values.

circRNA	cirexon	GM12878	HUVEC	HeLa-S3	HepG2	K562
**chr10:105197772|105198565**	105197772:105198565	0.167	n/a	n/a	0.495	0.167
**chr11:66006275|66006748**	66006275:66006748	0.077	n/a	n/a	n/a	n/a
**chr12:122218778|122219098**	122218778:122219098	0.451	n/a	n/a	n/a	n/a
**chr19:13039156|13039661**	13039156:13039477	0.25	n/a	n/a	n/a	0.091
**chr5:619105|620376**	619105:620376	0.125	n/a	0.121	n/a	n/a
**chr19:3660964|3661999**	3660964:3661999	n/a	0.419	n/a	n/a	n/a
**chr16:29917109|29917448**	29917109:29917448	n/a	n/a	0.316	0.667	n/a
**chr3:50145503|50145737**	50145503:50145737	n/a	n/a	0.312	n/a	n/a
**chr5:138994171|138994551**	138994171:138994359	n/a	n/a	0.25	n/a	n/a
**chr12:42604157|42604482**	42604157:42604482	n/a	n/a	n/a	0.1	n/a
**chr15:90431753|90432372**	90431753:90432372	n/a	n/a	n/a	0.346	n/a
**chr16:1859239|1859834**	1859239:1859834	n/a	n/a	n/a	0.333	n/a
**chr16:14720963|14721193**	14720963:14721193	n/a	n/a	n/a	0.111	n/a
**chr19:2137726|2138713**	2137726:2138713	n/a	n/a	n/a	0.408	0.238
**chr19:17387304|17387718**	17387304:17387718	n/a	n/a	n/a	0.849	n/a
**chr2:61749746|61753656**	61752405:61753656	n/a	n/a	n/a	0.385	n/a
**chr2:201721405|201721708**	201721405:201721708	n/a	n/a	n/a	0.625	n/a
**chr9:134312007|134314448**	134312007:134312282	n/a	n/a	n/a	0.049	n/a
**chr9:134381501|134381840**	134381501:134381840	n/a	n/a	n/a	0.385	0.035
**chr18:691068|691276**	691068:691276	n/a	n/a	n/a	n/a	0.009
**chr22:26853825|26854543**	26853825:26854543	n/a	n/a	n/a	n/a	0.118
**chr22:38208894|38209616**	38208894:38209616	n/a	n/a	n/a	n/a	0.5
**chr8:37623044|37623873**	37623044:37623264	n/a	n/a	n/a	n/a	0.215

Supplementary Table 10. **Samples of fruit fly used in this study for cirexon/exon detection.**

Sample	Data_amount	#BSJs	#Cirexons
virgin_female_eclosion_1_day_heads	17.6G	1034	1670
mated_female_eclosion_1_day_heads	18.2G	1139	1845
mated_male_eclosion_1_day_heads	16.9G	1196	1984
virgin_female_eclosion_4_day_heads	23.0G	1297	2071
mated_female_eclosion_4_day_heads	17.7G	1446	2340
mated_male_eclosion_4_day_heads	21.2G	1460	2399
virgin_female_eclosion_20_day_heads	16.0G	1835	3091
mated_female_eclosion_20_day_heads	16.9G	1354	2215
mated_male_eclosion_20_day_heads	19.3G	1995	3206
virgin_female_eclosion_4_day_ovaries	19.6G	323	451
mated_female_eclosion_4_day_ovaries	18.4G	379	515
mated_male_eclosion_4_day_testes	11.6G	353	500
mixed_males_and_females_eclosion_1_day_carcass	15.7G	464	689
mixed_males_and_females_eclosion_4_day_carcass	16.4G	759	1167
mixed_males_and_females_eclosion_20_day_carcass	15.2G	844	1298
mixed_males_and_females_eclosion_1_day_digestive_system	15.9G	532	706
mixed_males_and_females_eclosion_4_day_digestive_system	16.7G	805	1031
mixed_males_and_females_eclosion_20_day_digestive_system	19.3G	1004	1244
adult_midgut	22.8G	2447	2938
embryos_0-2_hr_after_egg_laying	23.2G	387	497
embryos_2-4_hr_after_egg_laying	14.1G	132	167
embryos_4-6_hr_after_egg_laying	14.5G	144	185
embryos_6-8_hr_after_egg_laying	13.0G	178	237
embryos_8-10_hr_after_egg_laying	10.0G	190	273
embryos_10-12_hr_after_egg_laying	11.2G	185	255
embryos_12-14_hr_after_egg_laying	19.9G	300	414
embryos_14-16_hr_after_egg_laying	12.4G	308	427
embryos_16-18_hr_after_egg_laying	13.1G	352	506
embryos_18-20_hr_after_egg_laying	23.6G	554	815
embryos_20-22_hr_after_egg_laying	12.7G	336	460
embryos_22-24_hr_after_egg_laying	21.1G	348	471
L3_stage_larvae_dark_blue_gut_PS1-2	36.1G	482	702
L3_stage_larvae_light_blue_gut_PS3-6	18.7G	278	391
L3_stage_larvae_clear_gut_PS_7-9	32.8G	637	940
third_instar_larvae_wandering_stage_digestive_system	16.6G	860	1096
third_instar_larvae_wandering_stage_CNS	30.2G	1020	1625
third_instar_larvae_wandering_stage_imaginal_discs	29.4G	446	664
third_instar_larvae_wandering_stage_salivary_glands	9.3G	190	275
WPP_salivary_glands	21.0G	498	718
larval_midgut	41.1G	6394	8387
Pupae_WPP_2d_CNS	13.9G	768	1240
S2-DRSC	9.6G	583	810
S2R	9.0G	401	532
S1	8.8G	268	377

ML-DmBG3-c2	9.3G	319	431
BG3-C2-R2-untreated	12.2G	438	598
BG3-C2-R2-5h_20E	11.4G	361	488
BG3-C2-R2-24h_20E	11.6G	398	540
BG3-C2-R3-5h_20E	12.5G	389	542
BG3C2_0hr_20E	19.9G	682	932
BG3C2_1hr_20E	19.8G	679	930
BG3C2_3hr_20E	9.2G	391	546
BG3C2_5hr_20E	10.0G	576	772
BG3C2_7hr_20E	19.3G	705	978
Kc167-R1-untreated	9.4G	427	634
Kc167-R2-untreated	11.6G	492	714
Kc167-R1-5h_20E	9.4G	378	554
Kc167-R2-5h_20E	9.2G	359	529
Kc167-R1-24h_20E	12.2G	644	943
Kc167-R2-24h_20E	9.9G	482	705
CME_W2	10.9G	415	575
CME_L1	10.8G	568	851
